# Recent strategies in synthetic food production for sustainable food security: applications and challenges

**DOI:** 10.3389/fnut.2026.1829214

**Published:** 2026-06-10

**Authors:** Pawan Kumar Kanaujia, Manikant Tripathi, Nagendra Kumar Rai, Rajat Pratap Singh, Monika Rao, Pradeep Kumar Singh, Pankaj Singh

**Affiliations:** 1 Department of Biotechnology, Faculty of Health and Life Sciences, Mahayogi Gorakhnath University, Gorakhpur, Uttar Pradesh, India; 2 Biotechnology Program, Dr. Rammanohar Lohia Avadh University, Ayodhya, Uttar Pradesh, India; 3 Department of Neurosciences, Cleveland Clinic, Cleveland, OH, United States; 4 Department of Biotechnology, Guru Ghasidas Vishwavidyalaya (A Central University), Bilaspur, Chhattisgarh, India; 5 Department of Biochemistry, Dr. Rammanohar Lohia Avadh University, Ayodhya, Uttar Pradesh, India

**Keywords:** cultured meat, dairy products, food security, microbial proteins, synthetic biology, synthetic foods

## Abstract

Due to rapid growth in population, instability in conventional agricultural production, and depletion of natural resources, currently, there is huge pressure to fulfill the demands for nutritious, safe, and cheaper food. Environmental changes have also impacted the livestock, forestry, aquaculture, and fisheries production in a variety of ways that may result in adverse health outcomes, trade disruption, compromised livelihoods, and negative economic effects. Recent technological development in synthetic food production has proven to be a transformative solution for the safe, nutritious, qualitative, and quantitative sustainable food requirements. The aim of this review is to address the need and explore recent technological innovations in cell, tissue, and stem culture technologies; genetically modified organisms (GMO); microbial fermentation technology; plant protein engineering using synthetic biology; artificial intelligence and digital technologies for the production of synthetic foods, such as cultured meat, artificial sweeteners, microbial proteins, novel fats, fortified and synthetic dairy products, and functional food ingredients with enhanced nutritional profiles and scalable production potential. The review will also explore the applications of synthetic foods in nutraceuticals, pharmaceuticals, food preservatives, and food additives, highlighting their significant role in improving human health. The findings of this review paper suggest that the development of synthetic food products could be a promising food resource toward resilient global food systems for the growing population. Although significant progress has been made in synthetic food production, it faces many challenges, such as high production costs, a lack of technological optimization, regulatory complexities, and safe use of synthetic foods. Also, for the successful commercialization of synthetic food products, there is a need to develop advanced technology to reduce the production cost and encourage legislative frameworks and public involvement.

## Introduction

In recent years, commercial production of synthetic food has emerged as a promising solution to address the challenges caused by the tremendous increase in the total population to fulfill the demand for a nutritious food supply (1, 2). It is expected that in the upcoming years, food demand will rise rapidly, which will require innovative approaches to fulfill this demand in a sustainable manner. Synthetic food production includes the production of food by using advanced biotechnological tools, such as cultivating animal stem cells, cell culture, tissue culture, fermentation, engineered microorganisms, and synthetic biology to produce specific functional ingredients, such as proteins, carbohydrates, and fats. It also offers viable alternative systems as compared to traditional food while ensuring nutritional adequacy and environmental sustainability (3, 4). It has been reported that currently, the total value of the global synthetic food market was USD 16 billion in 2022 and is projected to increase to USD 31 billion from 2022 to 2032 (5). Worldwide, North America is the leading hub for synthetic food production, and leading companies such as Symrise AG, Royal DSM N.V., Naturex SA, and Sensient Technologies are highly engaged in the synthetic food production. The global food system is currently undergoing a structural transition driven by converging pressures of population expansion, climate change, and resource depletion. Conventional agricultural systems, particularly livestock-based production, are increasingly recognized as environmentally unsustainable due to their disproportionate contributions to greenhouse gas emissions, land degradation, and water consumption. At the same time, nutritional inequalities persist across both developing and developed regions, manifesting as a dual burden of undernutrition and diet-related non-communicable diseases. These challenges underscore the urgent need for transformative food production strategies that decouple nutritional output from ecological constraints while maintaining affordability and scalability. Synthetic food products include the production of synthetic color, beverages, enzymes, dairy products, hydrocolloids, bakery products, antioxidants, flavor, snacks, emulsifiers, and fat replacers. Advancements in biotechnological approaches and increasing health concerns have motivated us for the production of synthetic foods that closely mimic the taste, texture, and nutritional composition of traditional animal- and plant-based products. Recent developments have shown that plant-based meat offers promising solutions to traditional meat production, as it has resource strain, environmental impact, health concerns, and ethical issues. However, there are significant differences in plant-based and animal-based meat in terms of appearance, texture, flavor, and nutrition. In the past, people in Asia produced meat substitutes from vegetables such as seitan from gluten, and tofu, tempeh from soy. However, these products failed to satisfy in terms of texture, flavor, and taste similar to meat. In 2002, research was conducted to produce cultured turkey and golden fish meat for its possible application in supplying astronauts’ food that was financed by the National Aeronautics and Space Administration (NASA) (6). The quality of cultured meat production has increased over time due to developments in cell culture techniques (7). However, due to high production costs, researchers are working on technological advancements for scaling up the industrialization and commercialization of cultured meat production (8). Companies are trying to develop dairy proteins with the help of microbial fermentation and advanced food processing techniques without conventional animals. Researchers are trying to develop microbial strains by using genetic engineering tools for qualitative and quantitative production of microbial products. Moreover, cellular agriculture has also emerged as a promising source for sustainable food production, involving animal cell culture in controlled environments to generate meat, poultry, and seafood products (9). Cellular agriculture mitigates the environmental impact of food production and fulfils the demand for protein-rich foods with optimized nutritional composition instead of traditional livestock farming (10). Although with these remarkable advancements, the commercial production of synthetic foods faces many challenges like lack of strict regulatory frameworks, consumer acceptance, critical factors like taste, cost, innovative technologies, and healthiness of synthetic food products that warrant careful consideration. It is essential to address these issues in order to fully explore the synthetic food’s potential as a sustainable and viable solution to the world’s food security problems. However, there is no doubt that the production of synthetic foods has an exciting future to transform the world food system for the coming generations. While significant progress has been made in the development of individual synthetic food technologies, existing literature often evaluates these systems in isolation, lacking a comprehensive framework that integrates their nutritional, environmental, economic, and functional dimensions. This fragmented approach limits the ability to assess their relative contributions to global food security and sustainable development goals. The present review addresses this gap by systematically categorizing major synthetic food types and critically analyzing their production technologies, nutritional profiles, sustainability metrics, and scalability challenges. By synthesizing cross-disciplinary insights, this study aims to provide a holistic perspective on the role of synthetic foods in shaping resilient and sustainable future food systems.

## Recent technological strategies for synthetic food production

Synthetic food production has emerged as a multidisciplinary response to the environmental, ethical, and nutritional challenges associated with conventional food systems (11). Livestock-based agriculture contributes substantially to greenhouse gas emissions, land degradation, freshwater use, and biodiversity loss, particularly in ruminant systems, where enteric methane and land use change dominate the climate footprint. At the same time, global demand for protein-rich foods continues to rise due to population growth, urbanization, and dietary shifts, intensifying pressure on already strained ecosystems. These dynamics have stimulated interest in “novel” or “alternative” protein sources—such as plant-based, microbial, insect-based, and cell-based proteins—as components of more sustainable and resilient food systems.

**Figure 1:**
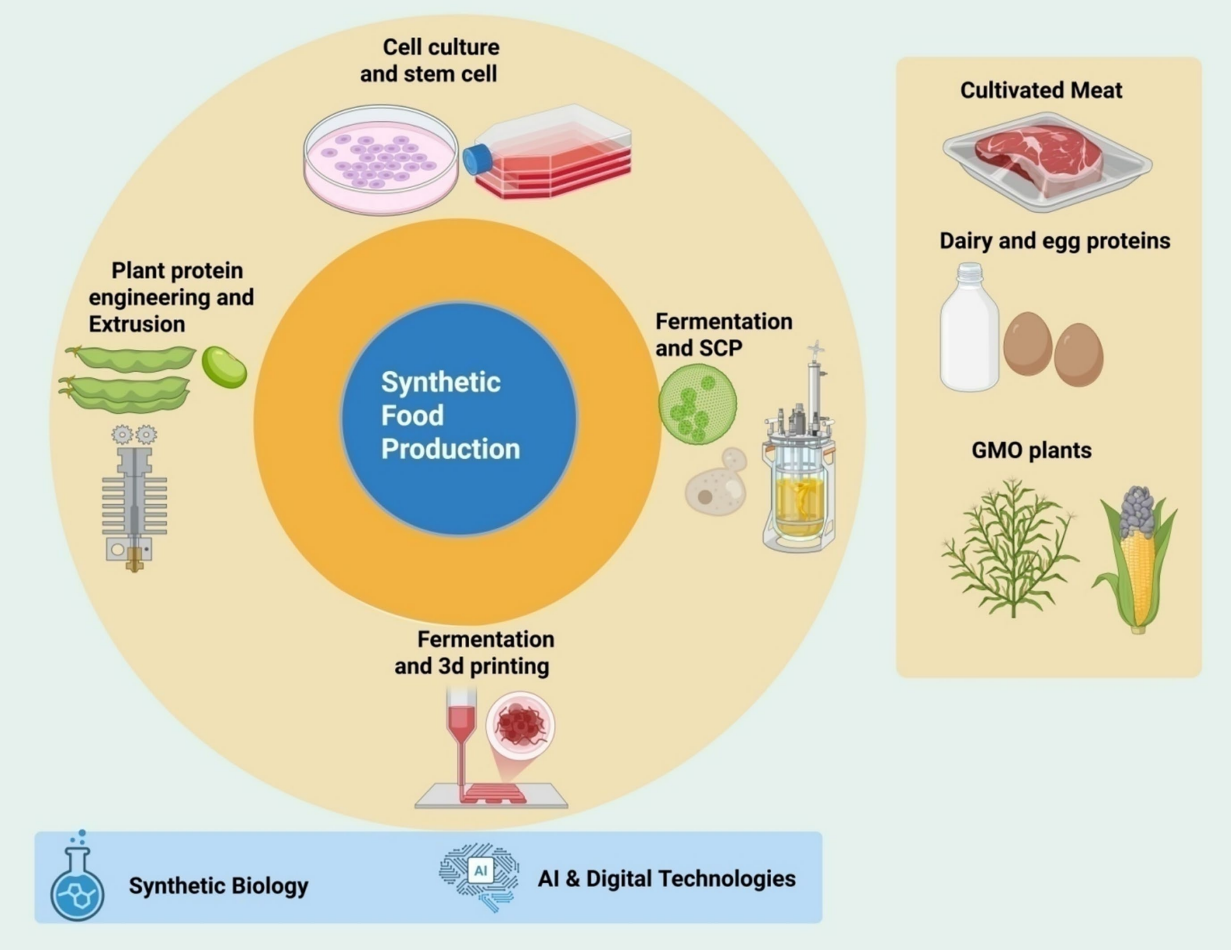
Converging technological strategies for synthetic food production. Schematic overview illustrating the major technological platforms that collectively enable synthetic food production, such as cell culture and stem cell technologies, fermentation and single-cell protein (SCP) production, plant protein engineering and extrusion, and tissue engineering with 3D food printing. These platforms converge toward the generation of diverse food products such as cultivated meat, animal-free dairy and egg proteins, and genetically modified plant-derived ingredients. Synthetic biology and artificial intelligence function as cross-cutting enablers that support strain engineering, process optimization, and scalable manufacturing across platforms.

Rather than representing a single technological solution, synthetic food production comprises a portfolio of converging approaches, such as animal cell culture, tissue engineering and 3D food printing, stem cell technologies, fermentation and single-cell protein production, plant protein engineering and extrusion, synthetic biology, and artificial intelligence-enabled process optimization. Each platform has distinct advantages and limitations in terms of scalability, cost, environmental performance, nutritional quality, and regulatory complexity. Recent reviews emphasize that these technologies are best understood within a system’s perspective, where different protein sources and processing methods are combined to address multiple objectives simultaneously, such as climate mitigation, land sparing, and improved nutritional profiles. Scenario analyses suggest that large-scale adoption of cellular agriculture and microbial proteins could significantly reduce agricultural land use and greenhouse gas emissions, although realized benefits will depend strongly on process efficiency, energy sources, and policy frameworks. Against this backdrop, the following subsections summarize key technological strategies currently shaping synthetic food production (Figure 1).

These technologies differ markedly in commercial readiness and scalability. Plant protein extrusion and biomass fermentation are commercially established, whereas cultivated meat remains largely pre-commercial. Precision fermentation has enabled products from companies such as Perfect Day and Remilk, although cost parity with conventional products remains unresolved. Single-cell protein has the longest track record, anchored by Quorn’s mycoprotein from *Fusarium venenatum* with over three decades of commercial history (12). Cultivated meat and tissue engineering remain pre-commercial in most markets: as of 2023, only Singapore and the United States had granted regulatory approval for cultivated chicken from GOOD Meat and Upside Foods, respectively, and in both cases at conditions far removed from mass-market scale (13, 14). Synthetic biology and artificial intelligence function as enabling layers that accelerate progress across all platforms without independently resolving the fundamental cost and scale barriers. The following subsections examine each technology with explicit attention to recent advances, commercial status, safety evidence, and outstanding limitations.

### Cell culture technology

Cell culture technology underpins the production of cultivated meat, seafood, and animal-derived fats by enabling ex vivo expansion and differentiation of animal cells under controlled conditions (15–17). Typically, a small biopsy is obtained from a donor animal, and relevant cell populations—such as muscle satellite cells, mesenchymal stem cells, or other myogenic and adipogenic progenitors—are isolated and propagated *in vitro* (18). Early studies focused on proof-of-concept demonstrations that muscle cells could be grown outside the animal and formed into simple tissues (19). More recent work has shifted toward developing robust, scalable bioprocesses that can deliver consistent biomass at competitive costs and within emerging regulatory frameworks.

A central area of progress is the stabilization and standardization of cell sources. Primary cells have finite proliferative capacity and often exhibit donor-to-donor variability, which complicates process control at an industrial scale (20). Current efforts therefore emphasize to establish well characterized cell banks with defined passage limits, reproducible growth kinetics, and predictable differentiation potential, such as conditionally immortalized lines and stem cell-derived progenitors tailored to specific species and tissue types (21). Such banks support more reliable manufacturing and facilitate regulatory review because cell identity and performance are documented and controlled.

Culture media composition remains the dominant operating cost in techno-economic assessments of cultivated meat. Historically, many protocols relied on fetal bovine serum, which may be ethically problematic, expensive, and poorly suited to large-scale food production. Consequently, there has been a major push toward serum-free and chemically defined media that use recombinant proteins, plant-derived hydrolysates, and small molecules instead of undefined animal serum. Recent work has demonstrated substantial reductions in growth factor requirements by optimizing receptor signaling, designing more efficient feeding strategies, and tailoring amino acid and lipid compositions to the metabolic needs of specific cell types (22). Media design is increasingly recognized as a crucial factor not only for cell proliferation and viability but also for nutritional quality because media components influence downstream features such as fatty acid profiles, micronutrient content, and the accumulation of bioactive compounds.

Scaling cell culture from laboratory flasks to industrial bioreactors is technically challenging because animal cells are shear sensitive and require narrow environmental tolerances. Stirred-tank and perfusion bioreactors are currently the most widely explored configurations, often using microcarriers or suspension-adapted lines to increase volumetric productivity while keeping shear stress within acceptable limits (23). Process analytical technologies such as online monitoring of pH, dissolved oxygen, key metabolites, and cell density are increasingly combined with advanced control strategies and “digital twins” to improve reproducibility, reduce contamination risk, and support rational scale-up. Techno-economic and life-cycle analyses identify volumetric productivity, media cost, energy demand, and contamination control as major determinants of both cost and environmental performance, underscoring the need to align biological optimization with engineering and energy system design. Cultivated meat raises important safety and regulatory concerns related to genetic stability, media composition, scaffold materials, and long-term cell culture effects (21, 24). Safety dossiers must characterize the entire production system: cell line provenance and genetic stability; culture media composition, such as any growth factors, cytokines, or undefined serum fractions; scaffold or carrier materials that may persist in the final product; and downstream processing aids (21, 25). Genetic instability in long-term or immortalized cell lines is a critical concern: chromosomal aberrations and epigenetic drift can alter protein expression profiles over successive production cycles, potentially generating neo-allergens or bioactive peptides absent from early safety evaluations (21). The allergenicity of novel scaffold materials—such as fungal chitosan, plant-derived zein, and bacterial nanocellulose—and of recombinant proteins expressed at high titers requires independent assessment, as these have no precedent in conventional food safety databases (26, 27). The FDA–USDA joint oversight framework in the United States and EFSA’s 2023 novel food guidance both mandate pre-market safety consultation with full characterization of production biology, but internationally harmonized standards for cultivated food safety assessment remain absent, creating unequal market access and slowing global commercialization (21). Regulatory oversight of cell-cultured foods has become increasingly defined. In the United States, the Food and Drug Administration (FDA) has established a pre-market consultation framework for foods made with cultured animal cells and publishes an inventory of completed consultations. In 2025, Australia and New Zealand, the Food Standards Australia New Zealand (FSANZ) had approved cell-cultured quail as a novel food, providing a clear regulatory precedent. These developments signal growing regulatory acceptance while emphasizing the need for robust safety, identity, and traceability data.

### Tissue engineering and 3D food printing

While cell culture provides biomass, tissue engineering and 3D food printing largely determine whether cultivated products resemble conventional meat in texture and sensory attributes (26, 28). The characteristic structure of meat, aligned muscle fibers, intramuscular fat, and a network of connective tissue governs mouth feel, juiciness, and cooking behavior. Replicating this hierarchical organization *in vitro* remains one of the most demanding objectives for cultivated meat technologies.

Tissue engineering strategies employ edible scaffolds that provide structural support and instructive cues for cell attachment, alignment, and maturation. Recent work has moved from biomedical scaffolds, such as collagen gels or synthetic polymers, toward food-grade materials based on plant polysaccharides, plant proteins, and fungal or algal matrices that are scalable, edible, and acceptable (26). These scaffolds must balance mechanical integrity with porosity to permit nutrient and oxygen diffusion, and their stiffness and microarchitecture are tuned to promote myotube formation and alignment. Some studies also apply mechanical or electrical stimulation during culture to enhance muscle phenotype, borrowing concepts from regenerative medicine but adapting them to food-grade materials and constraints.

Three-dimensional food printing and bioprinting add spatial precision by enabling controlled deposition of inks composed of plant proteins, hydrocolloids, fats, and, in some cases, cells. Most current food applications use printing to shape a cellular or low cell density matrix that can be produced at reasonable throughput and then combined with cultivated fat or cell-derived components to improve flavor and juiciness (29). Hybrid approaches—such as printing plant based fibrous matrices and subsequently incorporating cultured adipocytes or fat fractions—are especially attractive because they can markedly enhance sensory quality while greatly reducing the volume of expensive cultured cells required per product (30). Remaining challenges include maintaining structural integrity during cooking, reproducing realistic marbling patterns, and scaling 3D printing processes to industrially relevant volumes without high cost.

Despite rapid innovation, tissue engineering and 3D bioprinting have yet to demonstrate any product at a commercially relevant scale, and a candid assessment of current limitations is important. Structurally complex whole-cut products such as steak or chicken breast require vascularization-like nutrient and oxygen delivery networks to sustain cell viability beyond a few hundred microns of tissue thickness; no published study has achieved centimeter-scale thick structured tissue without perfusion bioreactor support (17, 26). From a safety perspective, plant-derived scaffolds. Such as decellularized spinach or pea protein gels, and fungal matrices such as bacterial nanocellulose, have not been fully assessed for allergenicity or toxicology under commercial-scale cell culture conditions, and regulatory novel food dossiers will need to address scaffold residues in finished products (26, 27). Commercially, Aleph Farms (Israel) and GOOD Meat (United States/Singapore) are among the most advanced companies pursuing structured cultivated meat, yet both have acknowledged cost-of-goods figures that remain far above mainstream retail viability (13, 31). A more tractable near-term pathway is the production of unstructured cultivated biomass—minced formats and fat co-ingredients—combined with plant protein matrices to achieve acceptable texture, a hybrid strategy that reduces tissue engineering complexity while enabling sensory improvement within current bioprocess and cost constraints.

### Stem cell culture

Stem cell technologies provide a renewable and standardized cell source for synthetic food production, enabling extensive expansion and controlled differentiation into muscle and adipose tissues while reducing reliance on repeated animal biopsies and improving batch consistency (16). In food applications, the primary goal of stem cell culture is not pluripotency itself, but robust proliferation, predictable lineage commitment, and long-term stability under industrial conditions. Recent studies have shown that differentiation efficiency can be enhanced while reducing dependence on costly cytokines by leveraging physical and metabolic cues, such as substrate stiffness, scaffold composition, mechanical stimulation, and energy metabolism modulation (32, 33). The development of suspension-adapted stem cell lines compatible with stirred-tank bioreactors further addresses scalability limitations associated with adherent cultures (34). Nevertheless, maintaining genomic stability during prolonged expansion, preventing unintended differentiation, and mitigating potential tumorigenic risks remain critical challenges for large-scale implementation. In addition, regulatory considerations and consumer perception may differ between pluripotent and tissue-specific stem cell–derived products, influencing technology selection and communication strategies.

In practice, most cultivated meat companies currently rely on satellite cells or mesenchymal stromal cells isolated from cattle, pigs, or poultry because these committed progenitors offer predictable myogenic or adipogenic differentiation without the regulatory and safety complexities associated with pluripotent stem cells (18, 35). Satellite cells derived from primary biopsies, however, display finite replicative capacity—typically 20–40 population doublings before senescence—making the development of stable, conditionally immortalized cell lines a critical unmet need for consistent industrial manufacturing (21). Strategies under active development to extend replicative lifespan include transient expression of telomerase reverse transcriptase (TERT) and cyclin-dependent kinase inhibitor suppression, though each introduces genetic modifications that complicate regulatory classification and consumer acceptance in markets with strict novel food definitions (27, 35). A particularly important but under-addressed safety dimension is the long-term genomic integrity of food production cell lines: unlike pharmaceutical biologics, where rigorous master cell bank qualification is mandatory. There is currently no internationally harmonized standard specifying acceptable karyotypic stability, mutation accumulation thresholds, or tumorigenicity testing requirements for cultivated food cell lines, a regulatory gap that urgently requires attention as commercialization accelerates (21, 24).

### Genetically modified organisms (GMO)

Genetic engineering plays a central role in many synthetic food technologies. Engineered microorganisms are widely used to produce enzymes, vitamins, amino acids, organic acids, sweeteners, and flavor compounds; in such cases, regulatory evaluation typically focuses on the safety and purity of the final ingredient rather than the genetic status of the production organism, provided that no viable genetically modified cells are present in the food (36). This paradigm has facilitated the widespread use of genetically modified yeasts and bacteria in fermentation-based production of food-grade ingredients. In cultivated meat systems, genetic modification may be applied to improve cell robustness, enhance nutrient utilization, or reduce dependence on exogenous growth factors (35). However, the use of genetically modified animal cells remains sensitive to regional regulatory frameworks and consumer perception, influencing the extent to which such strategies are adopted commercially.

The most significant recent advance in genetic engineering for synthetic food production is the adoption of CRISPR-Cas9 and related base- and prime-editing tools, which enable precise, scarless editing of production host genomes with substantially lower off-target risk than earlier nuclease platforms (37). In fermentation hosts, CRISPR-based metabolic rewiring has redirected carbon flux toward target molecules and introduced heterologous biosynthetic routes in production organisms, such as *Saccharomyces cerevisiae, Yarrowia lipolytica, Corynebacterium glutamicum,* and *Bacillus subtilis*, enabling higher yields of vitamins, amino acids, and functional lipids (37, 38). Impossible foods’ use of soy leghemoglobin expressed in *Pichia pastoris*—which received a “Generally Recognized as Safe” (GRAS) designation from the US FDA in 2019—illustrates the regulatory pathway for precision fermentation-derived ingredients where the GMO is the production host but is absent from the final food (36). A critical and frequently underappreciated distinction is between process-GMO applications, where the engineered organism produces a target molecule but is absent from the food, and product-GMO applications, where modified cells or tissues are themselves consumed. The latter face substantially higher regulatory hurdles and consumer resistance, particularly in the European Union, where the novel food regulation and GMO directive impose stringent pre-market authorization, traceability, and labeling requirements that have not yet been adapted to cultivated food contexts (13, 36). This regulatory asymmetry between jurisdictions represents a structural challenge for the global commercialization of GMO-enabled synthetic food technologies.

### Fermentation

Fermentation is currently the most industrially mature and scalable platform within the synthetic food landscape. It encompasses biomass fermentation, which produces edible microbial protein, and precision fermentation, which produces specific target molecules such as dairy proteins, egg proteins, enzymes, and lipids (39). Precision fermentation has enabled the commercial production of animal-identical whey and casein proteins that replicate the functional properties of conventional dairy ingredients, such as foaming, emulsification, and melting behavior. These proteins are now used in animal-free cheeses, yogurts, and beverages with sensory profiles similar to traditional products (38). Fermentation also plays an important supporting role in cultivated meat production by supplying growth factors, serum proteins, and other media components at scale (40). From a nutritional perspective, fermentation enables targeted fortification with vitamins and bioactive compounds, although claims must be supported by stability and bioavailability data in finished foods.

Several precision fermentation companies now provide concrete examples of both the promise and the current limitations of this platform. Perfect Day (United States) expresses bovine beta-lactoglobulin and alpha-lactalbumin in the filamentous fungus *Trichoderma reesei*, achieving gelation, foaming, and emulsification properties comparable to conventional whey, with ingredients incorporated in commercial ice cream, protein powders, and cream cheese in the United States (39). The Every Company (United States) produces ovalbumin and other egg white proteins in *Komagataella phaffii* (formerly *Pichia pastoris*) and has entered the food service sector, where functional equivalence to egg white is critical for binding and aeration in baked goods (38, 39). Remilk (Israel) uses a similar yeast-based system for beta-lactoglobulin and has obtained regulatory clearance in Israel, with applications pending in additional markets. Motif FoodWorks (United States) has developed HEMAMI™, a precision fermentation-derived myoglobin that imparts beef-like color and flavor to plant-based products, addressing one of the most persistent sensory gaps in that category. Despite these advances, techno-economic analyses indicate that animal-free dairy proteins currently cost 5 to 40 times more per kilogram than their conventional counterparts at current production scales, with cost parity projected only under scenarios of substantial bioreactor scale-up and media optimization not yet demonstrated industrially (22, 23, 41). This cost gap, rather than technical feasibility, represents the primary barrier to mass-market penetration and warrants priority attention in both academic research and public funding programmes.

### Single-cell protein (SCP) technology

Single-cell proteins derived from yeasts, filamentous fungi, bacteria, or microalgae represent a flexible class of alternative proteins with high productivity and a relatively small land footprint (42). SCP production exploits rapid microbial growth and can use carbon substrates that are not directly edible by humans, such as lignocellulosic hydrolysates, industrial effluents, or single-carbon feedstocks like methane or methanol (43). As a result, SCP can be produced with minimal land occupation and potentially low greenhouse gas emissions, depending on feedstock and energy sources. Recent advances focus on improving sensory quality and functional performance to expand SCP use in human foods. Controlled autolysis and fractionation can reduce nucleic acid content, alter cell wall structure, and produce protein concentrates with enhanced digestibility and milder flavor profiles (44). Blending SCP with plant proteins is increasingly common, as this can balance amino acid profiles and functional properties while diluting characteristic microbial flavors (43). SCP ingredients are being incorporated into hybrid foods, bakery products, and aquatic feeds, where they can partially replace fishmeal and soy without compromising performance. Key challenges include allergenicity assessment, regulatory approval for novel species and substrates, and ensuring consistent quality across different production facilities.

Commercially relevant SCP systems span a wide range of organisms, feedstocks, and regulatory maturity levels that merit specific discussion. Quorn’s mycoprotein, derived from *Fusarium venenatum* grown on glucose, is the most established human food SCP product globally, with a well-characterized safety profile accumulated over three decades of commercial sales and a favorable amino acid composition relative to most plant proteins (12). Solar Foods (Finland) produces Solein using the aerobic chemolithotroph *Cupriavidus necator*, which fixes CO_2_ using H_2_ and O_2_ in a process completely decoupled from agricultural land and photosynthesis; life cycle analysis indicates substantially lower land use and greenhouse gas emissions compared with conventional protein sources, though energy input requirements remain a key variable (45). Calysta (FeedKind) and UniBio (Uniprotein) produce protein biomass from *Methylococcus capsulatus* and related methanotrophs using natural gas as the sole carbon source, with current applications primarily in aquaculture feeds where regulatory thresholds are lower, though human food applications are under active development (46). Microalgae, such as *Chlorella vulgaris, Arthrospira platensis* (Spirulina), and *Haematococcus pluvialis,* occupy a distinct niche, combining protein production with high-value co-products such as astaxanthin and phycocyanin; they benefit from established regulatory approvals across the EU, United States, and Asia, but their high production costs and characteristic flavor profiles constrain use to niche functional food and supplement markets (42, 47). Across all SCP platforms, consumer acceptance remains an underexplored bottleneck: neophobia toward microbial food ingredients and regulatory labeling requirements for novel organisms can suppress demand regardless of nutritional or environmental performance, a dimension that the existing literature addresses only superficially.

### Plant protein engineering and extrusion

Plant protein engineering and high-moisture extrusion are currently the most commercially established synthetic food technologies (48). Plant proteins from soy, wheat, pea, fava bean, and other legumes are fractionated and processed to obtain ingredients with tailored solubility, emulsification, and gelation properties that support meat and dairy analogues. High moisture extrusion (HME) converts these blends into fibrous, meat-like structures by applying controlled heat, shear, and pressure in a twin screw extruder, followed by cooling in a die that promotes anisotropic alignment and phase separation (43). Recent studies have improved the understanding of how phase separation, protein cross-linking, and moisture gradients generate anisotropic textures, enabling more rational process optimization (49). Innovation continues to expand protein sources beyond soy and wheat, improve flavor through fermentation and enzymatic treatment, and structure fats to mimic animal fat melting behavior (50). Nutritional evaluation must consider both processing effects and final product composition under realistic cooking conditions.

Despite their commercial maturity, plant-based meat analogues face nutritional, sensory, and market challenges that receive insufficient critical attention in much of the published literature. From a nutritional standpoint, independent audits of supermarket plant-based products have documented sodium concentrations frequently exceeding 500–900 mg per 100 g serving—substantially above equivalent conventional meat products—reflecting the use of salt-dependent texturizing and flavoring strategies (51). Iron and zinc bioavailability from plant protein matrices is inherently limited by phytate and polyphenol complexation, and the iron in plant-based analogue products is typically non-haem iron with bioavailability as low as 5–12%, compared with 25–35% for haem iron in beef, raising genuine concerns about nutritional equivalence for vulnerable populations, such as pregnant women, adolescents, and those with low dietary diversity (52, 53). The protein quality of pea and wheat blends, while adequate by DIAAS criteria in some formulations, is sensitive to extrusion conditions and ingredient source, and is rarely reported for commercial products at the point of sale. Beyond nutritional composition, retail sales data from 2022 to 2024 consistently show declining unit volumes for plant-based meat in the United States and key European markets, with industry analyses attributing this to persistent price premiums of 1.5–2.5X the cost of conventional equivalents per gram of protein, unresolved sensory gaps particularly in fat mouth feel and aroma, and growing consumer wariness toward ultra-processed ingredient lists (14, 54). These converging pressures suggest that the field has prioritized production scale-up at the expense of nutritional optimization and consumer trust, and that future product development must integrate dietetics, food psychology, and sensory science alongside process engineering to achieve durable market relevance.

### Synthetic biology

Synthetic biology provides a rational framework for engineering microbial and cellular systems that underpin modern synthetic food production, particularly in precision fermentation, single-cell protein, and emerging cultivated meat platforms (39). By integrating standardized genetic parts, genome-scale metabolic models, and CRISPR-based genome editing, synthetic biology enables higher yields of target proteins and lipids, reduced by-product formation, and improved stress tolerance in industrial production hosts (37). In cultivated meat systems, similar approaches are being explored to reduce growth-factor requirements, enhance robustness under bioreactor conditions, and tailor lipid composition, although their application is constrained by regulatory frameworks and consumer acceptance when genetically engineered cells remain in the final product (27). Together, these advances position synthetic biology as a key enabling technology linking fermentation-based, cell-based, and plant-based synthetic food strategies.

Concrete examples illustrate both the power and the remaining limitations of synthetic biology in this field. Ginkgo Bioworks (United States) has applied automated strain engineering platforms combining high-throughput DNA assembly, combinatorial pathway construction, and machine learning-guided screening to accelerate production host optimization for food ingredient biosynthesis by 5–10-fold compared with traditional rational design (37). Impossible Foods applied synthetic biology to express soy leghemoglobin in a *Pichia pastoris* expression system, demonstrating how a single biosynthetically produced molecule can fundamentally transform the sensory profile of a plant-based product (39). In single-cell protein, metabolic engineering has rerouted carbon flux in *Yarrowia lipolytica* toward lipid and protein co-accumulation, producing biomass with a tailored fatty acid profile more closely resembling animal fat (37, 44). A critical limitation in food contexts, however, is that, unlike pharmaceutical or industrial biotechnology, target molecules must meet food-grade purity and safety standards, and the expression host must be excluded from the final product or itself be food-acceptable; this substantially narrows the choice of usable chassis organisms and requires additional downstream processing steps that add cost (38). Furthermore, intellectual property fragmentation—with foundational CRISPR technologies, genetic parts, and expression platform patents held by different entities—creates commercial access barriers that disproportionately disadvantage smaller research groups, a structural issue rarely discussed in scientific reviews but likely to shape the pace and geography of synthetic food innovation.

### Artificial intelligence and digital technologies

Artificial intelligence (AI) and digital technologies have emerged as cross-cutting enablers of synthetic food production, supporting optimization across cell culture, fermentation, extrusion, and formulation workflows (55). Machine learning approaches are increasingly used to optimize culture media, predict cell growth and differentiation outcomes, refine fermentation and extrusion parameters, and accelerate protein and enzyme design, such as sequence-to-function prediction and in silico screening for allergenicity and digestibility (39, 56). At the process level, digital twins and integrated sensor networks enable real-time monitoring, predictive control, and in silico scale-up of bioreactors and food processing systems, improving product consistency and reducing development risk. Beyond manufacturing, digital tools support life-cycle assessment, supply-chain traceability, and regulatory compliance, positioning AI-enabled data integration as a key determinant of scalability and sustainability in next-generation food systems.

Specific applications illustrate both the substantial promise and current practical constraints of AI in synthetic food production. In protein engineering, tools such as Alpha Fold2 (Deep Mind), ESM Fold (Meta AI), and RoseTTAFold have dramatically reduced the cost and time required to predict three-dimensional protein structures from sequence data, enabling in silico screening of novel protein variants for functional properties, such as thermal stability, emulsification, and gelation, before any wet-lab work is undertaken (56, 57). ProteinMPNN and related inverse folding models enable *de novo* design of protein sequences with target structures, opening the possibility of designing entirely novel food proteins with customized functional and nutritional profiles—an application directly relevant to precision fermentation and plant protein engineering (39, 56). In cell culture media optimization, Bayesian optimization and active learning frameworks have been applied to reduce growth factor concentrations while maintaining proliferation rates, with reported media cost reductions of 40–80% in proof-of-concept studies (22). Digital twin models of fermentation bioreactors use real-time sensor fusion and mechanistic-hybrid modeling to maintain optimal process trajectories during fed-batch and perfusion runs, reducing batch failure rates and improving yield consistency (55, 58). A critical limitation, however, is that AI models for synthetic food optimization are typically trained on sparse, proprietary datasets from single companies or processes, limiting generalizability and making benchmark comparisons across studies nearly impossible; establishing open, standardized datasets analogous to those in pharmaceutical bioprocessing is a prerequisite for realizing the full potential of AI in this field.

## Types of synthetic foods

**Figure 2:**
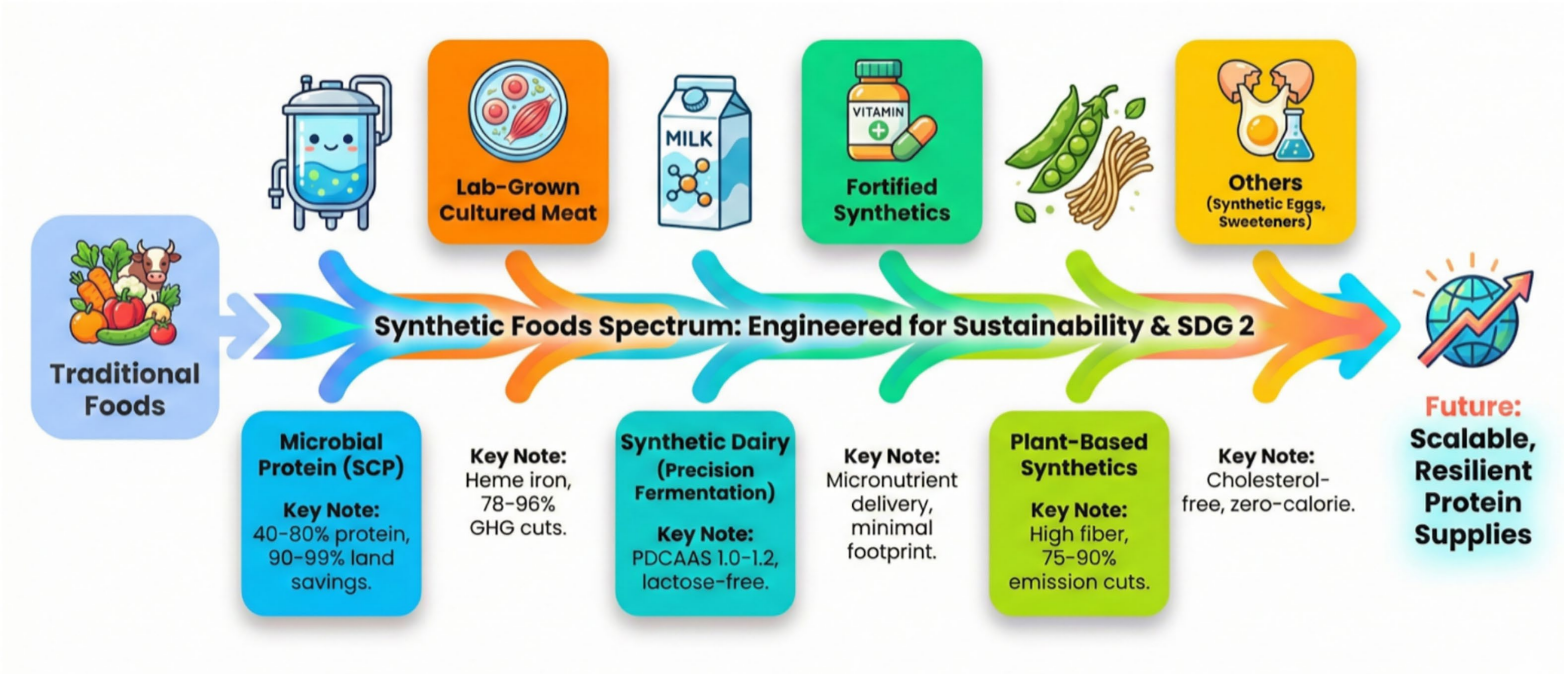
Classification of synthetic food categories and their role in sustainable nutrition. The figure illustrates the continuum from traditional foods to next-generation synthetic and engineered food systems designed to enhance sustainability and contribute to SDG-2 (Zero Hunger). Major categories include microbial protein or single-cell protein (SCP), lab-grown or cultured meat, synthetic dairy produced via precision fermentation, fortified synthetic foods for targeted micronutrient delivery, and plant-based synthetic alternatives. Key attributes such as protein yield, land and greenhouse gas (GHG) reductions, nutritional quality (e.g., PDCAAS), and health benefits (cholesterol-free, lactose-free) are highlighted for each category. Collectively, these approaches represent a transition toward scalable, resilient, and environmentally efficient protein and nutrient supplies for future food systems.

Synthetic foods represent a spectrum of engineered products derived from microbial, cellular, plant-derived, or chemical processes designed to emulate or surpass the nutritional, sensory, and functional properties of traditional foods, driven by imperatives for sustainability, resource efficiency, and global food security amid population growth and climate pressures (59). These technologies integrate bioprocessing innovations, such as precision fermentation, cell culture bioreactors, and formulation engineering, yet they grapple with inherent trade-offs in scalability, cost, sensory acceptance, and long-term health and environmental impacts (Table 1). The promising contributions to Sustainable Development Goal 2 (Zero Hunger) are through resilient protein supplies, and their adoption depends on biological variability, infrastructural demands, and socio-economic barriers within broader agri-food system transitions (59, 60). There are a number of available synthetic foods that are now widely used globally (Figure 2).

**Table 1:** Comparative overview of major synthetic food categories and their role in sustainable nutrition.

S. no.	Synthetic food type	Primary source/production technology	Nutritional characteristics	Key functional or health benefits	Environmental sustainability impact	Contribution to food security (SDG 2)	Economic feasibility and scalability	Major challenges/limitations	References
1.	Microbial protein (single-cell protein)	Fermentation of bacteria, yeast, or fungi on waste substrates (methanol, CO_2_) using bioreactors	40–80% protein; complete amino acid profile; rich in B-vitamins and minerals	High PDCAAS (~1.0); suitable for texturization; supports muscle repair	90–99% reduction in land and water use; low GHG emissions; waste valorization	Reduces protein deficiency in land-scarce regions; scalable for low-income populations	$2–10/kg; pilot-scale plants operational; industrial-scale bioreactors required	High nucleic acid content; consumer acceptance; regulatory approval	76, 77
2.	Lab-grown (cultured) meat	Stem cell proliferation in nutrient media with scaffolds in controlled bioreactors	Comparable to conventional meat; rich in protein, heme iron, vitamin B12; customizable fat profile	Sensory equivalence to meat; reduced saturated fats; improved cardiovascular profile	78–96% lower GHG emissions; 95–99% reduction in land and water use	Potential to subsidize nutrition in vulnerable populations via premium markets	$10–20/kg (declining); major scalability expected post-2030	High cost of growth media; ethical concerns; regulatory uncertainty	67, 69
3.	Synthetic dairy products	Precision fermentation of milk proteins or cell-cultured mammary cells	Highly bioavailable protein and calcium; lactose-free options available	Hypoallergenic alternatives; improved digestive tolerance	80–90% emission reduction; elimination of methane emissions	Fortified formulations help address childhood stunting and malnutrition	$15–50/kg; approaching cost parity with conventional dairy	Flavor complexity; microbial contamination risks	75
4.	Fortified synthetic foods	Microbial synthesis and encapsulation of vitamins and minerals in gels, powders, or bars	Targeted micronutrients with enhanced bioavailability	Effective against hidden hunger; improves immunity and development	Minimal land, water, and energy requirements	Enables large-scale micronutrient delivery in low-resource settings	<$2–3/kg; extrusion and spray-drying allow high scalability	Risk of over-fortification; shelf-life and stability issues	73, 142
5.	Plant-based synthetic foods	Extrusion, shearing, and structuring of pea, soy, or other plant proteins	High protein with dietary fiber; contains antioxidants and omega-3 fatty acids	Supports cardiometabolic health; cholesterol-free	75–90% reduction in land and water use compared to animal foods	Improves dietary diversity in vulnerable agroecological regions	$4–15/kg; mature industrial infrastructure	Amino acid imbalance; perception as ultra-processed foods	11, 63, 143
6.	Synthetic eggs	Fermentation-derived ovalbumin or plant-based hydrocolloid blends	High-quality protein; rich in choline; cholesterol-free	Excellent emulsification and foaming properties for baking	90–92% lower emissions than conventional egg production	Affordable protein alternative in water- and land-scarce regions	$3–10 per dozen equivalent; scalable fermentation systems	Foam stability challenges; protein yield limitations	64, 75
7.	Artificial sweeteners	Fermentation or chemical synthesis of non-caloric sweet compounds	Zero or low-calorie; high sweetness intensity	Glycemic control; weight management; diabetic-friendly	Reduces reliance on sugarcane monocropping	Enables palatable fortified foods without caloric load	$10–50/kg; highly scalable industrial processes	Potential gut microbiome effects; lingering aftertaste	77, 78

### Microbial protein

Single-cell protein (SCP) constitutes microbial biomass primarily from bacteria (e.g., *Methylophilus methylotrophus*), yeasts (*Saccharomyces cerevisiae*), filamentous fungi (*Fusarium venenatum*), or microalgae (*Spirulina platensis*) harvested after fermentation on inexpensive carbon sources like methane, molasses, or lignocellulosic wastes (38). Production platforms emphasize submerged fed-batch fermentation in stirred-tank bioreactors (volumes up to 100 m^3^), with process controls optimizing dissolved oxygen (20–30%), pH (5–7), and temperature (28–35 °C) for biomass yields of 50-–150 g/L dry weight; downstream steps include centrifugation, thermal nucleic acid hydrolysis, and spray-drying, addressing anti-nutritional factors (61). Nutritionally, SCP delivers 45–75% crude protein with a digestible indispensable amino acid score (DIAAS) of 0.9–1.1, rivaling casein or soy isolates, complemented by β-glucans for immunomodulation, B-vitamins (up to 10 mg/100 g thiamine), and minerals (iron, zinc at 20–50 mg/kg); however, fungal SCP has superior lipid oxidation stability but has potential purine overload risking hyperuricemia in gout-prone individuals (61, 62). Meta-analyses confirm 10–20% improvements in child growth metrics when SCP-fortified staples replace cereals in undernourished cohorts, underscoring functional attributes like emulsification in processed foods (62). Environmentally, life-cycle assessments (LCAs) project 85–95% reductions in greenhouse gas emissions (0.5–2 kg CO_2_e/kg protein), 99% less land, and 70–90% lower water footprints versus beef (20 kg CO_2_e/kg) (63). Economically, capital costs for 10,000-ton plants are approximately $50–100 million, with operating expenses at $3–8/kg yielding profitability at scale (>50,000 tons/year) by using CRISPR-engineered strains. This technology increases yields twofold; however, infrastructural retrofitting in developing regions and off-flavor masking impede commercialization (64). SCP plays an important role in SDG 2 resilience against supply–chain fluctuations and also strengthens food security by valorization of wastes in harsh environments. However, there are still issues related to regulatory approvals, safety measurements for microbial toxins/allergens, and bitterness of products. Comparatively, SCP outperforms cultured meat in energy efficiency but trails plant-based options in consumer familiarity (65).

### Lab-grown (cultured) meat

**Figure 3:**
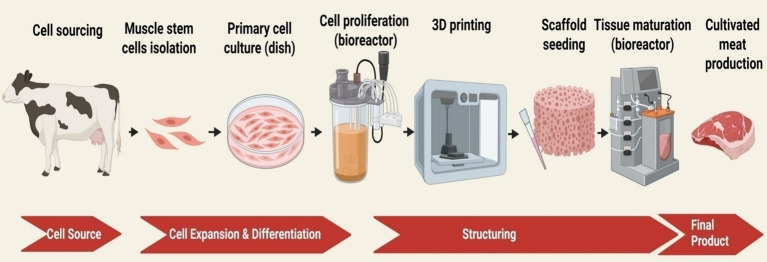
Simplified workflow of synthetic (cultivated) meat production. Cells obtained from a small animal biopsy are isolated and expanded under controlled conditions, proliferated in bioreactors, structured using scaffolds or 3D printing, and matured into meat-like tissue before harvesting and processing into final food products.

Cultured meat production, an alternative source of protein, offers a remarkable, sustainable, and slaughter-free solution to food security. The steps for lab-grown meat production include isolation of animal stem cells (myoblasts or fibroblasts) and expanded nutrition nurturing in bioreactors using chemically defined media [glucose, glutamine, and growth factors, such as fibroblast growth factor 2 (FGF-2)] followed by differentiation on edible scaffolds (i.e., collagen or fungal mycelia) with adipocytes co-culture for marbling meat culminating in structured tissues *via* mechanical or enzymatic maturation (Figure 3). Lab-grown cultured meat represents an innovative biotechnology solution for sustainable protein production (66, 67). Special systems like microcarriers and hollow fibers help achieve very high cell densities over 10^8^ cells/mL, while cells differentiate on edible scaffolds, such as collagen or fungal networks, often with added fat cells for marbling and realistic texture, finished by mechanical or enzyme treatments; however, challenges like physical stress on cells and waste buildup limit overall yields to just 5–15% efficiency (66, 67). This approach reduces traditional slaughter and farming, offering a controlled path to meat-like products.

Nutritionally, it closely matches the beef with around 22% protein, 5–15% fat, heme iron at 2–3 mg/100 g, and creatine levels of 300–500 mg/kg, higher levels of long-chain omega-3 fatty acids, and lower myoglobin level (68, 69). Digestion studies confirm bioavailability similar to conventional meat, with tenderness from muscle protein links, good satiety signals like GLP-1, and flavor influenced by fat breakdown, though stability of vitamins in low-oxygen conditions needs more research. Environmentally, it has been predicted that it will reduce 82–96% greenhouse gas production, require 92–99% less land, and up to 45% water savings versus beef or pork by using affordable media (70).

### Synthetic dairy products

Precision-fermented dairy products stand as a breakthrough in synthetic food technology by utilizing generally recognized as safe (GRAS) microbes like *Komagataella phaffii* yeast and *Aspergillus oryzae* fungi that are genetically modified to produce authentic bovine proteins, such as caseins (*α*s1, *β*, *κ*) and whey components (α-lactalbumin, β-lactoglobulin) at yields of 5–20 g/L in fed-batch fermenters running 72–120 h on glucose or glycerol feeds (71). This process cleverly addresses surging global dairy needs, especially for the 2 billion people affected by lactose intolerance, while tackling livestock emissions that contribute 3% to worldwide greenhouse gases. Following fermentation, proteins are purified to over 95% via ultrafiltration and chromatography and then blended with plant-based lipids to craft milks, cheeses, and wheys; innovative hybrid membrane bioreactors boost efficiency with continuous harvesting, reducing costs by 30–50% by avoiding downstream processing and ensuring steady output. Nutritionally, these dairy proteins excel with PDCAAS scores of 1.0–1.2, maintaining natural micelle structures for calcium bioavailability above 30% and offering lactose-free versions that ease digestion issues (42). Studies show that their nutritional value match with cow dairy in promoting muscle growth, with additional omega-3 boosts from algal oils to fight inflammation. Ultimately, they strengthen SDG 2 (Zero Hunger) by providing climate-proof, nutrient-rich options for lactose-sensitive populations, outperforming plant milks in protein superiority while fostering robust supply chains through targeted biotech infrastructure (72).

### Fortified synthetic foods

Fortified synthetic foods deliver a targeted biotechnology solution for nutrition security, embedding highly bioavailable micronutrients like NaFeEDTA iron and retinyl palmitate vitamin A into extruded grains, meat analogs, or beverages via hot-extrusion at 150–200 °C, vacuum coating, or liposomal encapsulation that shields against oxidation and Maillard reactions for 12–24-month shelf lives (63, 73). Dual-fortification with iron–zinc combinations harnesses synergistic chelation to maximize absorption, establishing these products as frontline defenses against hidden hunger in resource-limited regions facing intensifying malnutrition amid climate and population pressures, leveraging proven food engineering without complex bioreactors. Nutritionally, they boost hemoglobin 1–2 g/dL in anemic groups through 15–30% absorption rates, synergizing with protein matrices in analogs to tackle both macro- and micro-deficiencies, However, in the absence of ascorbic acid, phytates present in plant carriers reduce efficacy by 20–50%, while excessive fortification (more than 3 mg/day) in diverse diets poses a risk of vitamin A toxicity (74). Excellence in sustainability is achieved through precision dosing, preventing GDP losses from malnutrition of $3.5 trillion annually, increasing emissions by less than 5%, and reducing waste. Local agricultural analyses (LCAs) prefer it to crop biofortification in the short term, especially in cities or deserts, at a cost of $0.005–0.02 per serving, enabling programs like India’s 2024 fortified rice rollout to reduce stunting by 10–15% using existing varieties (73). Challenges include inequities in access, lack of label literacy, weak food matrix synergy compared to whole foods, and fortificant-induced flavor changes, as well as a lack of protein innovation, yet ethically supplement synthetic substances (75).

### Plant-based synthetic foods

Plant-based synthetic foods leverage extrusion-based biotechnology to recreate animal meat’s texture and nutrition from abundant plant isolates like pea (80% protein), soy, or mung bean, employing twin-screw extruders at 120–180 °C and shear rates of 500–1,000 s^−1^, enhanced by transglutaminase enzymes for cross-linking and methylcellulose binders that align proteins into fibrous, muscle-mimicking structures (76). Flavor profiles are refined with yeast extracts and leghemoglobin to evoke heme-derived umami and realistic browning, while high-moisture extrusion (HME) at 50–100% moisture integrates 20–30% fats for succulent burger patties with biomechanical fidelity nearing whole cuts, directly confronting protein supply strains from finite arable land. These deliver 18–26% protein exceeding eggs, plus 5–10 g/100 g fiber supporting digestion, though DIAAS scores of 0.65–0.92 constrained by lysine/methionine shortfalls require blends for completeness; pre-fermentation lifts bioavailability by 15%, offset by cardiovascular gains like 10% LDL drops despite isoflavone endocrine risks above 50 mg/day (11). Life-cycle assessments confirm 70–90% GHG cuts and 95% land savings over beef, moderated by soy deforestation potential and 10–20% processing emissions addressable via renewables; at $2-6/kg, they enable 10% global meat market disruption by 2030 through versatile extruders and low-capex modular plants (59, 63). Advancing SDG 2 (Zero Hunger), they facilitate affordable, resilient proteins eclipsing cultured meat economically, nearing single-cell neutrality integral to agri-food evolution (59).

### Synthetic eggs

Synthetic eggs provide an innovative, plant-based alternative to conventional eggs. They are made from a blend of pea and rice protein isolates, which provide 12% protein upon rehydration. They contain 0.5% gellan gum and sunflower lecithin lipids for ideal viscosity, which, along with the emulsification of the egg yolk, produces foaming up to 200–300%. These eggs are thermally denatured at 65–80 °C to mimic the behavior of natural eggs (64). Microbial-enhanced versions incorporate yeast hydrolysates to replicate protease activity for improved baking tenderness, while spray-drying produces stable powders with an 18-month shelf life at ambient conditions, effectively mitigating risks from avian influenza outbreaks and the land-intensive nature of poultry production in pursuit of diversified global protein sources. Nutritionally, these products offer 11% protein with a PDCAAS score of 0.95, serving as allergen-free options for 2% of the population but requiring fortification to replace missing natural choline (250 mg/egg equivalent) and B12; baking performance reaches 95% equivalence to traditional eggs, supported by enhanced lipid oxidation resistance that maintains superior flavor preservation during extended storage (73). Lifecycle benefits include an 80–95% reduction in land and water use compared to chicken eggs, increased resistance to pathogen disruption, and the promotion of circular economy practices through the use of plant byproducts (75).

### Artificial sweeteners

Artificial sweeteners represent a vital class of synthetic food ingredients crafted through targeted chemical and biotech processes, such as enzymatic bonding of aspartic acid and phenylalanine to create aspartame, chlorination of sucrose for sucralose, or fluorination for acesulfame-K, yielding 200–13,000 times the sweetness of sugar while remaining stable for baking and achieving over 99.5% purity through recrystallization techniques (77). These production methods, often run in efficient continuous reactors with purification steps like ion-exchange and crystallization, enable large-scale output with few byproducts, making them ideal palatability boosters for low-calorie synthetic beverages, baked goods, and precision-fermented products, effectively combating rising obesity rates and sugar-linked metabolic problems without diluting flavor profiles. Nutritionally, they contribute negligible calories (0.4–4 kcal/g) to support weight management and stable blood sugar within approved daily limits of 40–50 mg/kg. However, long-term studies reported that it has potential risks like glucose handling issues (RR 1.13–1.52) and alterations in gut bacteria, without adding benefits to proteins or other nutrients (78). From a sustainability perspective, manufacturing emissions are minimal (0.1 kg CO2e/kg) due to simplified synthesis, but persistent compounds with half-lives exceeding 100 days, such as sucralose, pose ecological concerns at small particle levels in water, highlighting the need for improved wastewater solutions (77).

## Applications of synthetic foods in industries

Synthetic foods are the major component of many industries as they impart color, provide flavor, and add nutritional value to food, such as vitamins and minerals. The use of synthetic foods is more popular as the different food-related industries are growing day by day. Various synthetic foods used in different industries are summarized below.

### Pharmaceutical and nutraceutical industry

#### Synthetic vitamins and minerals

Vitamins are organic compounds that are mainly plant-derived products. They are essential in minute amounts for the growth and development of body. Daily requirement of vitamins depends on diet as it cannot be produced at all or in adequate amounts in the body. Every vitamin holds a specific role, thus it can be irreplaceable (79). The synthesis of vitamin analogs at a lab scale is possible due to organic chemistry. Most of the synthetic vitamins used as supplements are coal tar-derived, petroleum extracts, and chemically processed (e.g., fish oils). The synthetic vitamins showed some vitamin activity with a diverse structure and lower cost than natural ones. Structure/function relationship is a key in human biology as it has a huge impact on storage, absorption, transport, and even the utilization of a molecule (80). For example, the synthetic form of tocopherol (vitamin E) is not identical to the natural form. The chemical reactions for the industrial synthesis of vitamins are not similar to the natural biosynthesis. The vitamin B complex (except vitamin B12) is completely synthesized in plants (81, 82). The laboratory analogues of plant vitamin B complex display greater chemical and biochemical diversity (82). Vitamin K is an essential micronutrient that is not synthesized by humans, and therefore it is obtained from vegan diets. Vitamin K is crucial for human health as it is involved in bone formation and blood clotting. Vitamin K3 (menadione), a distinct form of vitamin K, is an artificially produced form of vitamin K that is used for animal feed. It is a yellowish, crystalline molecule, frequently converted into an active form of vitamin K2 in the body of an animal (83, 84). Several vitamins exhibit antioxidant properties, which prevent tissues from oxidative stress in the body. Oxidative stress is an imbalance between the production of free radicals and their removal by antioxidants like vitamin C. The oxidative stress leads to many ageing and ageing-related diseases such as neurodegenerative and cardiovascular diseases, cancer, and diabetes (85, 86). The nutraceutical and pharmaceutical industries are constantly exploring and synthesizing new vitamins and antioxidant molecules to reduce the effects of oxidative stress. For example, the simil-microfluidic technique is used for the effective production of chitosan-coated nanoliposomes and nanoliposomal carriers encapsulating vitamin K2 and vitamin D3. It was found that the body cannot recognize whether a vitamin is natural or from laboratories for several reasons. Synthetic vitamins and minerals fortified staple food are the currently used strategy to overcome malnutrition and micronutrient deficiency in the population.

#### Amino acid mixtures

Amino acids play a crucial role in maintaining skeletal muscles in the body. For example, leucine, a branched-chain amino acid (BCAA) retards the loss of skeletal muscle mass by increasing the rate of muscle protein synthesis (87–89). However, the role of BCAA is reported in insulin sensitivity and glucose metabolism. The isoleucine increases glucose uptake and glucose transporter 4 membrane translocation. The amino acid arginine increases glucose uptake by promoting serine phosphorylation of Akt and helps insulin signaling in skeletal muscle under increased nitric oxide production (90). A novel amino acid mixture of glycine, isoleucine, and cystine is found useful in the improvement of glucose metabolism and insulin resistance (91). Thus, a synthetic and crystalline amino acids mixture is a useful alternative for soybean meal (diets for broiler) in chicken meat production (92).

#### Protein supplements

These are processed protein formulations and are extensively used to supplement protein intake to enhance muscle mass, recovery, and performance (93). Algal proteins and mycoproteins are the two microbial proteins that are widely used to cover a variety of proteins and protein ingredients. Protein supplements are available in various formulations, such as gummies, powders, protein bars, and ready-to-drink shakes. Most of the protein supplements are classified as hydrolysate, isolate, and concentrate-based protein (94, 95). The use of sports supplements is widely popular among the sports communities. In recent years, global consumption has increased (96). This increase among sports communities is suitable due to its promising ergogenic effects and its superior nutritional value. The ergogenic effects are shown by substances that were used to increase energy, prevent nutritional deficiencies, and improve performance among consumers (97, 98). Consumption of protein supplements offers improved body composition; improved endurance; and increased muscle mass, physical performance, and strength (99, 100). Whey protein supplements are wide spread formulation used by sports communities because of their quick retention and processing (101). A variety of protein supplements are used in sports nutrition and contribute to the fast growth of the pharmaceutical and nutraceutical industries. Plant and dairy are the popular protein sources, whereas cultured cell proteins, fermentation origin proteins, mycoproteins, and algal proteins are novel alternative sources of proteins that need to be introduced in the global market.

### Food processing industry

### Synthetic additives

Various synthetic additives are used in the food processing industry to attract consumers with their taste and satisfaction. Sweeteners are food additives that are used to replace sugar to sweeten and enhance the taste of foods, drinks, and pharma products. It is important to note that artificial sweeteners have no nutritional properties because they are not absorbed in the digestive system (102). Globally, artificial sweeteners are utilized as sugar substitutes in considerable amounts in food, snacks, beverages, drugs, etc. Aspartame, a dipeptide artificial sweetener, is frequently used in the food processing industry and is hydrolyzed in the digestive tract to its constituents, aspartic acid and phenylalanine (103). It is purchased under the brand names Natra, NutraSweet, and Equal. It was found that aspartame is 200-fold sweeter than sucrose, thus only a minute amount is required for sweetening drinks, juices, and foods. Another potent sweetener that is commercially used in the food processing industry is sodium cyclamate. It is commercially offered as a colorless and odorless sodium and calcium salt. Saccharin is the oldest and most popular artificial sweetener. Saccharin is utilized in products such as dairy products, carbonated beverages, juice, jams, table top sweeteners, chewing gum, puddings, desserts, and jellies (104). Saccharin is prepared by o-toluene sulfonamide upon electrochemical oxidation to carboxylic acid. The electrochemical oxidation is feasible because of various agents such as chromic acid and potassium permanganate (105). Another popular artificial sweetener, sucralose, is prepared from sucrose, which is 600-fold as sweet as the disaccharide, sucrose, with no calories. It is sold under the brand name Splenda since its approval by the FDA in 1998. It is utilized in bakery products, dairy products, carbonated beverages, desserts, jams, frozen desserts, juices, and other processed food products (106). Some novel technologies currently used to enhance the functionality of synthetic foods are nanoencapsulation, pulsed electric field (PEF), cold plasma, and high-pressure processing (HPP). These technologies contribute to extended shelf life, preserving food quality, and improving the distribution of additives in food components.

#### Synthetic food colors

Food colors are grouped as natural and synthetic in nature. Natural food colors are prepared from vegetables, seeds, fruits, barks, roots, and leaves without any chemical treatment. Synthetic food colors are additives used in the food and beverage industries as important formulations with the aim of satisfying consumer expectations. Experience of excellent food visuals even before taste is the expectation of the consumer (107, 108). Synthetic colors are used to boost the sensory experience of food for the consumer to match the product flavor (109). Mauveine was the first synthetic color synthesized by Sir William Henry Perkins in 1856 (110). Initially, chemical synthesis of colorants occurred from aniline, and it was simple to produce. These colors were of low cost, had better coloring properties, and mixed simply without imparting unnecessary flavors to food (111). Synthetic colors used as food additives were attractive because of their versatility and the ability to generate intense and uniform colors by controlling the number of chromophores on the main structural frame (112, 113). The commonly used food colors in food processing industries are Ponceau 4R, Tartrazine, Erythrosine, Carmoisine, Amaranth, Brilliant Blue, Allura Red, Sunset Yellow FCF, etc. (114). These synthetic colors are associated with some health concerns, such as allergies, mutagenic, and cytotoxic effects. To remove such adverse effects, synthetic biology and biotechnology, and encapsulation techniques permit the use of modified microorganisms for the production of natural colors as an alternative to artificial colors.

#### Synthetic flavors

Food served nowadays comes in a variety of tastes, colors, and aromas, which contribute to satiety and health. Aromas are extremely heterogeneous molecules due to the sources and chemical structures of foods. The term aroma is a central perception of all the senses of taste, smell, touch, and sight, but flavor allocates all the organolepticity of tasting. It is not very clear why food sensory characteristics coexist with aroma, appearance, and texture. The synthetic flavors are cost-effective alternatives to conventional foods. Artificial flavors are classified as synthetic or natural. An artificial flavor sometimes contains natural flavor derivatives. Some plant essential oils are commonly used as starting materials in the creation of flavor, whereas, some essential oils are concentrated by adsorption, partitioning, or distillation processes. Additionally, nanoencapsulation and microencapsulation processes have been used in food industry for preserving and enhancing the flavors (115, 116). The most widely utilized artificial flavoring compounds include phenolics, terpenoids, alcohols, esters, ketones, and pyrazines. They are used in soups and stocks, canned soups, dry soups, frozen soups, sauces, seasonings, and marinades, meat products, baked goods and bakery products, fruit-based drinks, fermented beverages, dairy products, fermented milk, etc. The Food and Drug Administration (FDA, United States) carries out several studies to check the overuse of artificial flavoring, which is of fully chemical origin (117).

#### Fat substitutes

Fat replacers are substances that are used to replace some or all of the fat present in food; however, they give the food texture, taste, etc., similar to the original whole-fat food. Fat in food adds volume, food flavor, and mouth feel. They serve up two main purposes. They reduce the quantity of fat and the calorie content of the food. A health-aware population looks for means to improve nutritional habits without giving up satisfaction (118, 119). These people explored low-fat diets as they relate to a reduced risk of obesity and chronic heart disease (120). High fiber and low fat foods can significantly reduce the risk of obesity, colon cancer, cardiovascular diseases, etc. (121). A product is said to be reduced calorie or reduced fat if it contains 25% less calories or 25% fat that is smaller than the regular version of the food product. Fat replacers are mainly classified into two groups, fat mimetics and fat substitutes. Fat mimetics are the ingredients that are unlike the chemical structures of fat. They are chemically either of carbohydrate or protein origin. Fat substitutes are constituents that possess a chemical structure almost similar to fats and offer similar physicochemical properties (122). Fat replacers include carrageenan, cellulose, powdered cellulose, dextrins, polydextrose, gums, pectin, modified whey protein, isolated soy protein, sucrose polyester, salatrim, caprenin, mono and diglycerides, which are commonly used in food processing industries, etc. Some methods/techniques, such as Oleogels, 3D food printing, HPP, enzymatic interesterification, and emulsion templates, are used to produce fat replacers.

#### Merits and demerits of synthetic food

With an increasing population and limited resources, the task of providing adequate nutrition has become a global priority. To overcome the food shortage, synthetic foods with appropriate nutritional value would play a significant role. Various synthetic foods such as plant-based, cultured meat, foods prepared using microbes-based fermentation technology, and foods based on microalgae are gaining attention in recent times (Table 2). However, these synthetically developed foods have their own merits and demerits. Therefore, it becomes very important to understand strategies for the detection and evaluation of these synthetic foods to assure appropriate supply of nutrition with utmost safety. Synthetic food is artificially developed using chemicals. These foods can tackle the challenges like food scarcity, high costs, and short shelf life by providing higher quality, the possibility of nutrient fortification, affordability, and higher shelf life (123). When considering the inclusion of synthetic food in our diets, it is important to understand the potential health consequences linked to synthetic foods. These artificial additives are commonly found in processed foods, which can pose health risks. Warner (124) discussed the long-term health effects of the use of food additives. They reported that high intake of non-caloric sweeteners is linked to different types of health impacts, such as cardiovascular disease, depression in adults, and childhood obesity. These chemicals require a proper assessment of quantitative limits.

**Table 2:** Companies that produce synthetic foods by using different microorganisms/cell systems.

Synthetic food	Engineered microorganism/cell system used	Production system	Company	Characteristics features
Genetically modified rennet enzyme	*Aspergillus niger, Escherichia coli*, *Kluyveromyces lactis*	Microbial fermentation	Multiple dairy industries	Cheese production without animal-derived rennet
Whey protein	*Trichoderma reesei*	Fermentation	Perfect Day	Production of animal-free β-lactoglobulin
Spirulina protein products	*Arthrospira platensis*	Photobioreactor cultivation	Various companies	Sustainable source of nutritious protein
Egg white proteins	*Komagataella phaffii,* *Pichia pastoris*	Fermentation	The EVERY Company	Production of animal-free egg proteins and ovalbumin
Meat production	Chicken cells + algal biopolymers	3D bioprinting and scaffold systems	Biokraft Foods	Production of synthetic meat similar to conventional meat
Cheese proteins	Yeast strains	Precision fermentation	Formo	Production of animal-free cheese proteins
3D-printed cultivated fish fillet	Fish muscle and fat cells	3D bioprinting + cell culture	Umami Bioworks and Steakholder Foods	Synthetic seafood production
Lactose-free milk proteins	Yeast/fungi	Large-scale fermentation	Remilk	Produces animal-free dairy-identical proteins
Plant-based meat and flavor	*Pichia pastoris*	Precision fermentation	Impossible Foods	Production of plant-based meat and meat-like aroma and flavor
Mycoprotein (Quorn products)	*Fusarium venenatum*	Biomass fungal fermentation	Quorn Foods	Production of protein-rich fungal meat
Cultivated chicken	Chicken satellite stem cells	Animal cell culture in bioreactors	Upside Foods	One of the first approved cultivated meat products

### Benefits of synthetic foods

#### Controlled and improved nutrition

Synthetic foods are produced and formulated with balanced nutritional requirements that ensure uniform nutritional quality compared to natural foods. With modern biotechnological approaches and food science, the quality of food can be improved. Synthetic biology can be applied to provide edibles, designed with improved quality of nutrients, for example, vitamins, proteins, and lipids with a better calorific value (125). In another work, Tyagi et al. (126) discussed the foods that were prepared using synthetic biology, which served as an important parameter for improving the quality of dairy products, providing a better way to overcome the storage of food with an improved shelf life and monitored packing. Carotenoids are effective compounds in the yeast cell factory that are being extensively used as feed additives. Synthetic biology techniques are being used to improve the level of beta-carotene production in yeast (127). Nutrients, such as amino acids, are designed and developed especially with improved health perspectives, which also widen the consumption of feedstocks and offer high-quality food synthesis (128). The creation of genetically modified organisms (GMOs) has had a remarkable effect in the improvement not only in fruits and vegetables but also in the dairy industry. The use of recombinant DNA technology for improving the production of milk in cows and the production of chymosin from a bacterial system has been discussed by Kennedy et al. (129). With controlled and improved quality of essential nutrients, the dietary and health requirements would be achieved, which provides overall health benefits to individual.

#### Enhanced food security

Food security is very important for people’s healthy lives. Synthetic foods with extended shelf-life help in food security. In the current scenario, a broad range of methodologies are being used for the detection and quantification of possible threats that can be present in the synthetic food products. Lab-grown proteins and fortified synthetic foods can support the growing populations, which ultimately might contribute to food security (130). Artificial intelligence may contribute to the enhancement of food security by using advanced systems like predictive analytics algorithms, robotic inspection systems, sensor networks, and supply chain optimization tools to guarantee food security at a global level (131). Some other detection techniques, such as enzyme-linked immunosorbent assay (ELISA), polymerase chain reaction (PCR), chromatography, biosensors based, microfluidic technology, and isothermal amplification are also being used to ensure enhanced food security of synthetic edibles (132).

#### Enhanced shelf life

The enhanced shelf life of synthetic food products is significant as it supplies the increasing demand for safe and nutritionally balanced food. The need for synthetic foods with improved nutritional values has also shifted people’s preference for food options with a prolonged shelf life, without causing any food-borne problems (133). In a study, researchers discussed anti-microbial packing systems that play an important role in extending the shelf life of food products, by extending the lag phase and slowing down the growth rate of microorganisms, leading to an extended shelf life and maintaining food safety (134). Moreover, advancements in food technologies have significantly helped in increasing the shelf life while maintaining its quality and nutrition (135, 136). These studies suggest that the enhancement of shelf life in synthetic foods is crucial for future food requirements as well as their storage.

### Demerits of synthetic foods

#### Regulatory and safety challenges

The rapid development of synthetic foods has introduced significant regulatory and safety challenges for global food systems. The consumption of highly processed foods serves as a pro-inflammatory dietary factor to human health. In a recent study, researchers discussed that food colors like Red 40, present in diets with high amounts of fat, have been highlighted as a cause of DNA damage and colonic inflammation in mice (89).

#### Digestibility and bioavailability

Despite various benefits of synthetic foods, their digestibility and bioavailability are challenges in their applications as compared to natural food products. In a study, Fardet (137) discussed that during the manufacturing of synthetic foods, the excess processing and chemical modifications can denature biomolecules like proteins and alter lipid structures, making them less accessible to enzymes involved in digestion. In another recent study, Muncke et al. (138) discussed that the digestibility and bioavailability of synthetic foods are questionable because chemical substances, such as bisphenols and phthalates, have been pinpointed for causing regressive effects during prenatal and perinatal development. Research also suggests that chemicals, particularly emulsifiers, might have harmful effects on the intestine, which may result in decreased absorption and bioavailability of nutrients present in synthetic foods (139).

#### Future prospects and challenges

Synthetic edibles have been designed and modified to play a more advanced role in providing improved and enriched nutrition for better health and management of diseases. There are various future challenges regarding the availability of these foods, keeping in mind the toxins released by the microorganisms, a compromise between safety and quality, the economic concerns, and enhanced availability of nutrients while protecting the limited natural resources (140). One of the most significant obstacles is going to be the opinion of the customer, regarding the presence of engineered microorganisms in the edibles in a certain proportion. The requirement of an alternative source for preservation, renewable packaging, and resource availability for the production of biomass, and the need for inexpensive feed stocks, which can be produced without much effort and not disturbing the classical food chain, is another hurdle in count (141). So, different types of challenges, such as technical and regulatory, during synthetic food production should be carefully tackled with the help of bio-based and advanced molecular biology techniques to ensure future food security.

## Conclusion

Synthetic foods represent a transformative shift in the design and production of nutritional systems, offering the potential to decouple food production from traditional agricultural constraints. As demonstrated across microbial protein, cultured meat, precision-fermented dairy, plant-based analogs, and fortified synthetic products, these technologies collectively address critical dimensions of food security, such as resource efficiency, nutritional adequacy, and supply resilience. However, their impacts are not uniform, and each category presents distinct trade-offs between scalability, environmental performance, cost, and consumer acceptance. Future food security is one of the most important concerns and a global challenge. To tackle this challenge, developing synthetic foods can be a promising solution. These foods are designed to provide targeted human health with balanced nutrients. There are different strategies involved in its development, such as chemical and microbial fermentation technology, while there are still limitations in using these foods because of their bioavailability, costs, concerns about digestibility, and efforts from all stakeholders to address ethical, regulatory, and socio-economic considerations. There is a need for research and technological innovations to assure cost-effective, good-quality, safe, and nutritionally balanced synthetic foods for future food security. In conclusion, synthetic food represents a compelling solution to the complex challenges facing our global food system. By harnessing the power of technology and innovation, we can generate a more sustainable, resilient, and equitable food supply chain. Among the evaluated systems, microbial protein and plant-based synthetic foods currently offer the most immediate and scalable solutions due to their relatively low production costs, established processing infrastructure, and favorable environmental profiles. In contrast, cultured meat and precision-fermented dairy represent high-fidelity alternatives with significant long-term potential but remain constrained by economic and technological barriers, particularly in large-scale bioprocessing and energy requirements. Fortified synthetic foods, while not primary protein sources, play a critical complementary role in addressing micronutrient deficiencies and enhancing public health outcomes. Looking forward, the successful integration of synthetic foods into global food systems will depend on advances in bioprocess optimization, renewable energy integration, regulatory harmonization, and consumer trust-building. Interdisciplinary innovation, coupled with policy support and equitable access strategies, will be essential to ensure that these technologies contribute meaningfully to Sustainable Development Goal 2 (Zero Hunger). Ultimately, rather than replacing conventional agriculture entirely, synthetic foods are likely to function as complementary systems within a diversified food production framework, enabling a more resilient, sustainable, and nutritionally optimized future.

## References

[1] Lv X Wu Y Gong M Deng J Gu Y Liu Y . Synthetic biology for future food: research progress and future directions. Future Foods. (2021) 3:100025. doi: 10.1016/j.fufo.2021.100025

[2] Singh P Tripathi M Singh PK Monika Rayeen F Pathak N. In: Proceedings of the 5th International Electronic Conference on Foods, editor. Recent Advancement in Commercial Production of Synthetic Food for Global Food Security: Future Prospective and Challenges. Basel: MDPI (2024)

[3] Chen R Ren S Li S Zhou H Jia X Han D . Synthetic biology for the food industry: advances and challenges. Crit Rev Biotechnol. (2024) 45:–47. doi: 10.1080/07388551.2024.2340530, 38797660

[4] Rout S Srivastav PP. Recent trends in the production of proteins by precision fermentation for improving the quality and attributes of food: role of genetic engineering towards next generation of food production. Microbe. (2025) 8:100551. doi: 10.1016/j.microb.2025.100551

[5] Pulidindi K (2023). Synthetic Food Market Size by Product (Colour, Enzymes, Hydrocolloids, Flavour, Fragrances, Antioxidants, Emulsifiers, Fat Replacers), by Application (Beverages, Dairy and Frozen Products, Bakery and Confectionery, Others) and Forecast. 2023–2032 (2427: GMI2427). Global Market Insights, Inc.. Available online at: https://www.gminsights.com/industry-analysis/synthetic-food-market (Accessed May 06, 2026).

[6] Benjaminson MA Gilchriest JA Lorenz M. In vitro edible muscle protein production system (MPPS): stage 1, fish. Acta Astronaut. (2002) 51:879–89. doi: 10.1016/s0094-5765(02)00033-4, 12416526

[7] Guan X Lei Q Yan Q Li X Zhou J Du G . Trends and ideas in technology, regulation and public acceptance of cultured meat. Future Foods. (2021) 3:100032. doi: 10.1016/j.fufo.2021.100032

[8] Ye Y Zhou J Guan X Sun X. Commercialization of cultured meat products: current status, challenges, and strategic prospects. Future Foods. (2022) 6:100177. doi: 10.1016/j.fufo.2022.100177

[9] Soice E Johnston J. How cellular agriculture systems can promote food security. Front Sustain Food Syst. (2021) 5:1–13. doi: 10.3389/fsufs.2021.753996

[10] Kardas M Staśkiewicz-Bartecka W Kołodziejczyk A. Cultured meat reformulation: health potential and sustainable food challenges-narrative review. Compr Rev Food Sci Food Saf. (2025) 24:e70262. doi: 10.1111/1541-4337.70262, 41063486 PMC12508633

[11] Poore J Nemecek T. Reducing food’s environmental impacts through producers and consumers. Science. (2018) 360:987–92. doi: 10.1126/science.aaq0216, 29853680

[12] Whittaker JA Johnson RI Finnigan TJA Avery SV Dyer PS. The biotechnology of Quorn mycoprotein: past, present and future challenges. Fungal Biol Biotechnol. (2020) 7:1. doi: 10.1186/s40694-019-0079-531921433

[13] GFI (Good Food Institute) State of the Industry Report: Cultivated Meat and Seafood Good Food Institute (2023). Available online at: https://gfi.org/resource/cultivated-meat-eggs-and-dairy-state-of-the-industry-report/ (Accessed May 02, 2026).

[14] Santo RE Kim BF Goldman SE Dutkiewicz J Biehl EMB Bloem MW . Considering plant-based meat substitutes and cell-based meats: a public health and food systems perspective. Front Sustain Food Syst. (2020) 4:134. doi: 10.3389/fsufs.2020.00134

[15] Post MJ. Cultured meat from stem cells: challenges and prospects. Meat Sci. (2012) 92:297–301. doi: 10.1016/j.meatsci.2012.04.008, 22543115

[16] Bhat ZF Morton JD Mason SL Bekhit AEDA Bhat HF. Technological, regulatory, and ethical aspects of in vitro meat: a future slaughter-free harvest. Compr Rev Food Sci Food Saf. (2019) 18:1192–208. doi: 10.1111/1541-4337.12473 33336995

[17] Gu H Kong Y Huang D Wang Y Raghavan V Wang J. Scaling cultured meat: challenges and solutions for affordable mass production. Compr Rev Food Sci Food Saf. (2025) 24:e70221. doi: 10.1111/1541-4337.70221, 40635127 PMC12241508

[18] Nunes OBDS Buranello TW Farias FA Rosero J Recchia K Bressan FF. Can cell-cultured meat from stem cells pave the way for sustainable alternative protein? Curr Res Food Sci. (2025) 10:1–12. doi: 10.1016/j.crfs.2025.100979 PMC11878651 40040753

[19] Dumont NA Bentzinger CF Sincennes MC Rudnicki MA. Satellite cells and skeletal muscle regeneration. Compr Physiol. (2015) 5:1027–59. doi: 10.1002/cphy.c140068 26140708

[20] Wang Y Tong X Niu J Chen X Zheng W Ma X. The key technologies and main challenges for the engineering production of animal cell meat cultured in bioreactors. Food Prod Process and Nutr. (2025) 7:57. doi: 10.1186/s43014-025-00327-y

[21] Bennie RZ Ogilvie OJ Loo LSW Zhou H Ng SK Jin A . A risk-based approach can guide safe cell line development and cell banking for scaled-up cultivated meat production. Nat Food. (2025) 6:25–30. doi: 10.1038/s43016-024-01085-9, 39753758

[22] Quek JP Gaffoor AA Tan YX Tan TRM Chua YF Leong DSZ . Exploring cost reduction strategies for serum free media development. NPJ Sci Food. (2024) 8:107. doi: 10.1038/s41538-024-00352-0, 39709448 PMC11663224

[23] Humbird D. Scale-up economics for cultured meat. Biotechnol Bioeng. (2021) 118:3239–50. doi: 10.1002/bit.27848, 34101164 PMC8362201

[24] EFSA Panel on Nutrition, Novel Foods and Food Allergens (NDA). Scientific opinion on cultivated meat: safety considerations for cell culture media, scaffolds and other production components. EFSA J. (2023) 21:e07951. doi: 10.2903/j.efsa.2023.7951 37151988 PMC10157499

[25] McClements DJ Grossmann L. A brief review of the science behind the design of healthy and sustainable plant-based foods. NPJ Sci Food. (2021) 5:17. doi: 10.1038/s41538-021-00099-y, 34083539 PMC8175702

[26] Seibert GA Feddern V Bastos APA Kumar A Verruck S. Trends in non-animal scaffolds for cultured meat structuration. NPJ Sci Food. (2025) 9:208. doi: 10.1038/s41538-025-00429-4, 41109903 PMC12535591

[27] Garbin VP Ferreira GA Olegário JC Olegário JDC Poniewas L de Macedo REF. Animal-derived components in cultivated meat research and their alternatives. NPJ Sci Food. (2025) 10:7. doi: 10.1038/s41538-025-00656-9, 41392297 PMC12789690

[28] Ben-Arye T Shandalov Y Ben-Shaul S Landau S Zagury Y Ianovici I . Textured soy protein scaffolds enable the generation of three-dimensional bovine skeletal muscle tissue for cell-based meat. Nat Food. (2020) 1:210–20. doi: 10.1038/s43016-020-0046-5

[29] Rinshana PF Murugesan B Kim YH Alaguthevar R Rhim JW. Advances in 3D food printing technology: innovation and applications in the food industry. Food Sci Biotechnol. (2025) 34:403–21. doi: 10.1007/s10068-024-01779-7, 39944672 PMC11811361

[30] Maharjan S Yamashita C Lee CP Villalobos Zepeda A Michel Farias AK Duarte Rivera A . 3D bioprinting of plant and animal cell-based hybrid food. Nat Commun. (2025) 16:6935. doi: 10.1038/s41467-025-61996-4, 40721583 PMC12304182

[31] Moritz MSM Verbruggen SEL Post MJ. Alternatives for large-scale production of cultured beef: a review. J Integr Agric. (2015) 14:208–16. doi: 10.1016/S2095-3119(14)60935-3

[32] Stout AJ Zhang X Letcher SM Rittenberg ML Shub M Chai KM . Engineered autocrine signaling eliminates muscle cell FGF2 requirements for cultured meat production. Cell Rep Sustain. (2024) 1:100009. doi: 10.1016/j.crsus.2023.100009

[33] Kolodkin-Gal O Dash R Rak R. Probiotic cultivated meat: bacterial-based scaffolds and products to improve cultivated meat. Trends Biotechnol. (2024) 42:269–81. doi: 10.1016/j.tibtech.2023.09.002, 37805297

[34] Kwok CK Ueda Y Kadari A Günther K Ergün S Heron A . Scalable stirred suspension culture for the generation of billions of human induced pluripotent stem cells using single-use bioreactors. J Tissue Eng Regen Med. (2018) 12:e1076–87. doi: 10.1002/term.2435, 28382727

[35] Kim S Jeong Y Jo H Park YG Moon SH. Cultured meat: advances in stem cell biology, tissue engineering, and bioprocess optimisation for scalable and sustainable production—a review. Int J Food Sci Technol. (2025) 60:vvaf220. doi: 10.1093/ijfood/vvaf220

[36] Bawa AS Anilakumar KR. Genetically modified foods: safety, risks and public concerns-a review. J Food Sci Technol. (2013) 50:1035–46. doi: 10.1007/s13197-012-0899-1, 24426015 PMC3791249

[37] Lee SY Kim HU. Systems strategies for developing industrial microbial strains. Nat Biotechnol. (2015) 33:1061–72. doi: 10.1038/nbt.3365, 26448090

[38] Verma K Duhan P Pal D Verma P Bansal P. Precision fermentation for the next generation of food ingredients: opportunities and challenges. Future Foods. (2025) 12:100750. doi: 10.1016/j.fufo.2025.100750, 38826717

[39] Nielsen MB Meyer AS Arnau J. The next food revolution is here: recombinant microbial production of Milk and egg proteins by precision fermentation. Annu Rev Food Sci Technol. (2024) 15:173–87. doi: 10.1146/annurev-food-072023-034256, 38134386

[40] Singh S Yap WS Ge X Min VLX Choudhury D. Cultured meat production fuelled by fermentation. Trends Food Sci Technol. (2022) 120:48–58. doi: 10.1016/j.tifs.2021.12.028

[41] GFI (Good Food Institute) State of the Industry Report: Fermentation Good Food Institute (2023b). Available online at: https://gfi.org/resource/fermentation-state-of-the-industry-report/ (Accessed May 05, 2026).

[42] Alves SJF Pires EBE Alexandre MADS Santos CCADA Martin JGP Campelo PH . Single-cell proteins as alternative sources of proteins and nutrients. Food Res Int. (2025) 214:116631. doi: 10.1016/j.foodres.2025.116631, 40467219

[43] Zhuang Z Wan G Lu X Xie L Yu T Tang H. Metabolic engineering for single-cell protein production from renewable feedstocks and its applications. Adv Biotechnol (Singap). (2024) 2:35. doi: 10.1007/s44307-024-00042-8, 39883267 PMC11709146

[44] Balagurunathan B Ling H Choi WJ Chang MW. Potential use of microbial engineering in single-cell protein production. Curr Opin Biotechnol. (2022) 76:102740. doi: 10.1016/j.copbio.2022.102740, 35660478

[45] Smetana S Sandmann M Schebesta S Leiber F Mathys A. Sustainability of alternative proteins: life cycle assessment of Solein production via power-to-food concept. Future Foods. (2023) 7:100215. doi: 10.1016/j.fufo.2022.100215

[46] Øverland M Tauson AH Shearer K Skrede A. Evaluation of methane-utilising bacteria products as feed ingredients for monogastric animals. Arch Anim Nutr. (2010) 64:171–89. doi: 10.1080/17450391003691534, 20578647

[47] Linder T. Making the case for edible microorganisms as an integral part of a more sustainable and resilient food production system. Food Secur. (2019) 11:265–78. doi: 10.1007/s12571-019-00912-3

[48] Sui X Zhang T Zhang X Jiang L. High-moisture extrusion of plant proteins: fundamentals of Texturization and applications. Annu Rev Food Sci Technol. (2024) 15:125–49. doi: 10.1146/annurev-food-072023-034346, 38359947

[49] Zhang X Shen A Zhang Z Zhang T Jiang L Zhou W . Advancing molecular understanding in high moisture extrusion for plant-based meat analogs: challenges and perspectives. Food Chem. (2024) 460:140458. doi: 10.1016/j.foodchem.2024.140458, 39029364

[50] Jafarzadeh S Qazanfarzadeh Z Majzoobi M Sheiband S Oladzadabbasabad N Esmaeili Y . Alternative proteins: a path to sustainable diets and environment. Curr Res Food Sci. (2025) 9:100882. doi: 10.1016/j.crfs.2024.100882 PMC11827122 39958969

[51] Curtain F Grafenauer S. Plant-based meat substitutes in the flexitarian age: an audit of products on supermarket shelves. Nutrients. (2019) 11:2603. doi: 10.3390/nu11112603, 31671655 PMC6893642

[52] Fresán U Mejía MA Craig WJ Sabaté J Rajaram S. Meat analogues from plant and fungal sources and relevance of their zinc, iron and phytate content for the dietary transition to sustainable diets. Nutrients. (2019) 11:2458. doi: 10.3390/nu1111245831615154

[53] Parodi A Leip A De Boer IJM Slegers PM Ziegler F Temme EHM . The potential of future foods for sustainable and healthy diets. Nat Sustain. (2018) 1:782–9. doi: 10.1038/s41893-018-0169-2

[54] GFI (Good Food Institute) State of the Industry Report: Plant-Based Meat, Seafood, Eggs, and Dairy Good Food Institute (2023c). Available online at: https://gfi.org/resource/plant-based-meat-eggs-and-dairy-state-of-the-industry-report/

[55] Ibarra-Muñoz LA Acosta GGR García RM Mata LYA Martínez JDS Oyervides LM . Artificial intelligence in the food and bioprocess industries: addressing food security challenges. Food Hum. (2025) 5:100818. doi: 10.1016/j.foohum.2025.100818

[56] Yang K Zachary W Arnold FH. Machine-learning-guided directed evolution for protein engineering. Nat Methods. (2019) 16:687–94. doi: 10.1038/s41592-019-0496-6, 31308553

[57] Jumper J Evans R Pritzel A Green T Figurnov M Ronneberger O . Highly accurate protein structure prediction with AlphaFold. Nature. (2021) 596:583–9. doi: 10.1038/s41586-021-03819-2, 34265844 PMC8371605

[58] Liné A Pinsach J Gódia F. Digital twins in bioprocess development: current state and future perspectives. Biotechnol Adv. (2022) 60:108016. doi: 10.1016/j.biotechadv.2022.108016, 35781046

[59] Alae-Carew C Green R Stewart C Cook B Dangour AD Scheelbeek PFD. The role of plant-based alternative foods in sustainable and healthy food systems: consumption trends in the UK. Sci Total Environ. (2022) 807:151041. doi: 10.1016/j.scitotenv.2021.151041, 34673070 PMC8724617

[60] More TA Shaikh Z Ali A. Artificial sweeteners: a review. Biosci Biotechnol Res Asia. (2021) 18:227–37. doi: 10.13005/bbra/2910

[61] Blackstone NT Pavlova A Trinidad KR Nikkhah A Sinke P Heller M . Guidelines for environmental life cycle assessment of cultivated meat. Int J Life Cycle Assess. (2025) 30:2943–63. doi: 10.1007/s11367-025-02562-4

[62] Das K Roy P Tiwari RKS. Biofortification of rice: an impactful strategy for nutritional security: current perspectives and future prospect. Plant-Based Diet. (2023):1–28. doi: 10.5772/intechopen.110460

[63] BIOVIT. Synthetic vs Natural: Start-up Targets Fortification Category Disruption. Food Navigator (2023). Available online at: https://www.foodnavigator.com/Article/2023/10/06/Synthetic-vs-natural-Start-up-targets-fortification-category-disruption-with-organic-plant-derived-nutrients/ (Accessed May 10, 2026).

[64] Choi KR Ahn DH Jung SY Lee YH Lee SY. Microbial lysates repurposed as liquid egg substitutes. NPJ Sci Food. (2024) 8:35. doi: 10.1038/s41538-024-00281-y, 38898024 PMC11187216

[65] Salgueiro MJ Zubillaga M Lysionek A Caro R Weill R Boccio J. Fortification strategies to combat zinc and iron deficiency. Nutr Rev. (2002) 60:52–8. doi: 10.1301/00296640260085958, 11852970

[66] Post MJ Levenberg S Kaplan DL Genovese N Fu J Bryant CJ . Scientific,sustainability and regulatory challenges of cultured meat. Nat Food. (2020) 1:403–15. doi: 10.1038/s43016-020-0112-z

[67] Risner D . Lab-grown meat carbon Footprint Worse than Retail Beef. UC Davis Food News (2023). Available online at: https://www.ucdavis.edu/food/news/lab-grown-meat-carbon-footprint-worse-beef (Accessed May 10, 2026).

[68] Tuomisto HL de Mattos MJ. Environmental impacts of cultured meat production. Environ Sci Technol. (2011) 45:6117–23. doi: 10.1021/es200130u, 21682287

[69] Anjum M. Khan M. Khalid S. Ali N. Al-Hinaai M. M. Global outlook on the meat market and alternatives: plant-based and cultivated meat challenges, developments, and opportunities. Curr Res Nutr Food Sci (2026) 14:86–116. Available online at: https://bit.ly/3MrPeDL (Accessed May 09, 2026).

[70] Ishaq A Irfan S Sameen A Khalid N. Plant-based meat analogs: a review with reference to formulation and gastrointestinal fate. Curr Res Food Sci. (2022) 5:973–83. doi: 10.1016/j.crfs.2022.06.001, 35721393 PMC9198813

[71] Green Queen Media. (2023). Animal-Free Milk: What Precision Fermentation Dairy LCAs Tell Us. Available at: https://www.greenqueen.com.hk/animal-free-milk-precision-fermentation-dairy-lcas/ (Accessed May 02, 2026).

[72] Li YP Ahmadi F Kariman K Lackner M. Recent advances and challenges in single cell protein (SCP) technologies for food and feed production. NPJ Sci Food. (2024) 8:66. doi: 10.1038/s41538-024-00299-2, 39294139 PMC11410949

[73] World Bank. Food Fortification: A Global Overview. World Bank Group. (2020). Available online at: https://www.worldbank.org/en/topic/agriculture/brief/food-security-update (Accessed May 02, 2026).

[74] Eastham JL Leman AR. Precision fermentation for food proteins: ingredient innovations, bioprocess considerations, and outlook—a mini-review. Curr Opin Food Sci. (2024) 58:101194. doi: 10.1016/j.cofs.2024.101194

[75] Zidon-Eyal R. Precision fermentation dairy: sustainability and scalability. Trends Biotechnol. (2024) 42:345–58. doi: 10.1016/j.foodres.2024.115527

[76] Abedfar A Abbaszadeh F Mardiha F. A review on the importance of producing single-cell protein (SCP) from agricultural by-products and waste. Chem Biomol Eng. (2025) 10:8–15. doi: 10.11648/j.cbe.20251001.12

[77] Wei Y Ji B. The health effects of artificial sweeteners: towards personalized quantification and prediction through gut microbiome. Eco-Environ Health. (2023) 2:89–91. doi: 10.1016/j.eehl.2023.05.003, 38074993 PMC10702885

[78] Li J Li X Li Y Liu H Wang Q. Artificial sweeteners in wastewater treatment plants: a systematic review of global occurrence, distribution, removal, and degradation pathways. J Hazard Mater. (2025) 494:138644. doi: 10.1016/j.jhazmat.2025.138644, 40393290

[79] Thiel R. Natural vitamins may be superior to synthetic ones. Med Hypotheses. (2000) 55:461–9. doi: 10.1054/mehy.2000.1090, 11090291

[80] Kiyose C Muramatsu R Kameyama Y Ueda T Igarashi O. Biodiscrimination of alpha-tocopherol stereoisomers in humans after oral administration. Am J Clin Nutr. (1997) 65:785–9. doi: 10.1093/ajcn/65.3.785, 9062530

[81] Kennedy DO. B vitamins and the brain: mechanisms dose and efficacy—a review. Nutrients. (2016) 8:68. doi: 10.3390/nu8020068, 26828517 PMC4772032

[82] Lindschinger M Tatzber F Schimetta W Schmid I Lindschinger B Cvirn G . Bioverfügbarkeiteinesnatürlichen versus einessynthetischen Vitamin-B-Komplexes und derenAuswirkungen auf metabolischeProzesse. MMW Fortschr Med. (2020) 162:17–27. doi: 10.1007/s15006-020-0230-4 32189314

[83] Jan YH Richardson JR Baker AA Mishin V Heck DE Laskin DL . Vitamin K3 (Menadione) redox cycling inhibits cytochrome P450-mediated metabolism and inhibits parathion intoxication. Toxicol Appl Pharmacol. (2015) 288:114–20. doi: 10.1016/j.taap.2015.07.023, 26212258 PMC4579064

[84] Drug Bank. Menadione. (2005).Available online at: https://go.drugbank.com/drugs/DB00170 (Accessed May 04, 2026).

[85] Pincemail J Defraigne JO Cheramy-Bien JP Dardenne N Donneau AF Albert A . On the potential increase of the oxidative stress status in patients with abdominal aortic aneurysm. Redox Rep. (2012) 17:139–44. doi: 10.1179/1351000212Y.0000000012, 22732574 PMC6837517

[86] Pincemail J Kaci M Cheramy-Bien Defraigne JO Meziane S. Electrochemical methodology for evaluating skin oxidative stress status (SOSS). Diseases. (2019) 7:40. doi: 10.3390/diseases7020040 31137870 PMC6631060

[87] Duan Y Li F Li Y Tang Y Kong X Feng Z . The role of leucine and its metabolites in protein and energy metabolism. Amino Acids. (2016) 48:41–51. doi: 10.1007/s00726-015-2067-1 26255285

[88] Martinez-Arnau FM Fonfria-Vivas R Buigues C Castillo Y Molina P Hoogland AJ . Effects of leucine administration in sarcopenia: a randomized and placebo-controlled clinical trial. Nutrients. (2020) 12:932. doi: 10.3390/nu12040932, 32230954 PMC7230494

[89] Zhang Q Chumanevich AA Nguyen I Chumanevich AA Sartawi N Hogan J . The synthetic food dye, red 40, causes DNA damage, causes colonic inflammation, and impacts the microbiome in mice. Toxicol Rep. (2023) 11:221–32. doi: 10.1016/j.toxrep.2023.08.006, 37719200 PMC10502305

[90] Jobgen WS Fried SK Fu WJ Meininger CJ Wu G. Regulatory role for the arginine–nitric oxide pathway in metabolism of energy substrates. J Nutr Biochem. (2006) 17:571–88. doi: 10.1016/j.jnutbio.2005.12.001, 16524713

[91] Miura K Hasumura M Kitahara Y Nishitani S Yamada Y. A novel amino acid mixture containing isoleucine, glycine, and cystine improves insulin sensitivity with restoring mitochondrial oxygen consumption in C2C12 myotubes. Funct Foods Health Dis. (2025) 15:205–16. doi: 10.31989/ffhd.v15i4.1582

[92] Selle PH de Paula Dorigam JC Lemme A Chrystal PV Liu SY. Synthetic and crystalline amino acids: alternatives to soybean meal in chicken-meat production. Animals. (2020) 10:729. doi: 10.3390/ani10040729, 32331461 PMC7222841

[93] Samal JRK Samal IR. Protein supplements: pros and cons. J Diet Suppl. (2018) 15:365–71. doi: 10.1080/19390211.2017.1353567, 28937838

[94] Lagrange V Whitsett D Burris C. Global market for dairy proteins. J Food Sci. (2015) 80:A16–22. doi: 10.1111/1750-3841.12801, 25757893

[95] Carter BG Drake MA. Invited review: the effects of processing parameters on the flavor of whey protein ingredients. J Dairy Sci. (2018) 101:6691–702. doi: 10.3168/jds.2018-14571, 29885888

[96] Colmenero MV Martinez-Sanz JM Navarro AN Ortiz-Moncada R Hurtado JA Baladia E. Variables used in questionnaires about ergonutritionals supplements intake. Nutr Hosp. (2015) 32:556–72. doi: 10.3305/nh.2015.32.2.8373, 26268083

[97] Maughan RJ Greenhaff PL Hespel P. Dietary supplements for athletes: emerging trends and recurring themes. J Sports Sci. (2011) 29:S57–66. doi: 10.1080/02640414.2011.587446, 22150428

[98] Rodriguez RF Mirta Crovetto M Andrea Gonzalez A Nikol Morant C Francisco Santibanez T. Nutritional supplement intake in gymnasium: consumer profile and charateristics of their use. Rev Chil Nutr. (2011) 38:157–66. doi: 10.1016/j.nut.2009.06.021, 20004078

[99] Petrenko AS Ponomareva MN Sukhanov BP. Regulation of food supplements in the European Union and its member states. Part 2. Vopr Pitan. (2014) 83:52–7. Available at: https://pubmed.ncbi.nlm.nih.gov/25549474/25549474

[100] de Antunano NPG Manonelles P Redondo RB Fernandez CC Bonafonte LF Aurrekoetxea TG . Suplementosnutricionales para eldeportista. Ayudasergogénicaseneldeporte—2019. Med Deport. (2019) 36:7–83. Available at: https://archivosdemedicinadeldeporte.com/articulos/upload/Doc-consenso-ayudas-2019.pdf).

[101] Haun CT Vann CG Mobley CB Roberson PA Osburn SC Holmes HM . Effects of graded whey supplementation during extreme-volume resistance training. Front Nutr. (2018) 5:84. doi: 10.3389/fnut.2018.00084, 30255024 PMC6141782

[102] Kroger M Meister K Kava R. Low-calorie sweeteners and other sugar substitutes: a review of the safety issues. Compr Rev Food Sci Food Saf. (2006) 5:35–47. doi: 10.1111/j.1541-4337.2006.tb00081.x

[103] Trefz F de Sonneville L Matthis P Benninger C Lanz-Englert B Bickel H. Neuropsychological and biochemical investigations in heterozygotes for phenylketonuria during ingestion of high dose aspartame (a sweetener containing phenylalanine). Hum Genet. (1994) 93:369–74. doi: 10.1007/BF00201660, 8168806

[104] Weihrauch M Diehl V. Artificial sweeteners—do they bear a carcinogenic risk? Ann Oncol. (2004) 15:1460–5. doi: 10.1093/annonc/mdh256, 15367404

[105] Bennett C Dordick JS Hacking AJ Cheetham PS. Biocatalytic synthesis of disaccharide high intensity sweeterner sucralose via a tetrachlororaffinose intermediate. Biotechnol Bioeng. (1992) 39:211–7. doi: 10.1002/bit.260390213, 18600933

[106] Whitehouse CR Boullata J McCauley LA. The potential toxicity of artificial sweeteners. AAOHN J. (2008) 56:251–61. doi: 10.3928/08910162-20080601-02, 18604921

[107] Burrows A. Palette of our palates: a brief history of food coloring and its regulation. Compr Rev Food Sci Food Saf. (2009) 8:394–408. doi: 10.1111/j.1541-4337.2009.00089.x

[108] Rohrig B. Eating With Your Eyes: The Chemistry of Food Colorings. Chem Matters (2015), 5–7. Available online at: https://teachchemistry.org/chemmatters/october-2015/eating-with-your-eyes-the-chemistry-of-food-colorings (Accessed May 05, 2026).

[109] McAvoy SA. Global Regulations of Food Colors. The Manufacturing Confectioner (2014), 77–86. Available online at: https://www.iacmcolor.org/wp-content/uploads/2014/09/PMCA-2014-McAvoy.pdf (Accessed May 04, 2026).

[110] Downham A Collins P. Colouring our foods in the last and next millennium. Int J Food Sci Technol. (2000) 35:5–22. doi: 10.1046/j.1365-2621.2000.00373.x

[111] Nida S Umar ZN Ismat S. Survey on the use of synthetic food colors in food samples procured from different educational institutes of Karachi city. J Trop Life Sci. (2013) 3:1–7. doi: 10.11594/jtls.03.01.01

[112] Singh PK Singh P Singh RP Singh RL. Biodecolorization of azo dye acid blue 113 by soil bacterium *Klebsiella variicola* RMLP1. J Ecophysiol Occup Health. (2021) 21:64–71. doi: 10.18311/jeoh/2021/27108

[113] Singh PK Singh RP Singh P Singh RL. Efficient decolorization of dye acid blue 113 by soil bacterium *Bacillus subtilis* RMLP2. Toxicol Int. (2021) 28:267–78. doi: 10.18311/ti/2021/v28i3/27736

[114] Bachalla N. Identification of synthetic food colors adulteration by paper chromatography and spectrophotometric methods. IAIM (2016), 3: 182–191. Available online at: http://iaimjournal.com/

[115] Panakkal EJ Kitiborwornkul N Sriariyanun M Ratanapoompinyo J Yasurin P Asavasanti S. Production of food flavoring agents by enzymatic reaction and microbial fermentation. Appl Sci Eng Prog. (2021) 14:297–312. doi: 10.14416/j.asep.2021.04.006

[116] Salunkhe AA Tamboli FA Zade MS Ghadge YR Kore MD More AD. Natural flavoring agents used in pharmaceutical industry. Int J Pharm Chem Anal. (2023) 10:150–5. doi: 10.18231/j.ijpca.2023.027

[117] Singh N Sudha ML. Natural food flavours: a healthier alternative for bakery industry-a review. J Food Sci Technol. (2024) 61:642–50. doi: 10.1007/s13197-023-05782-4, 38410266 PMC10894155

[118] Kostas G. Low-fat and delicious: can we break the taste barriers? J Am Diet Assoc. (1997) 97:S88–92. doi: 10.1016/s0002-8223(97)00738-4, 9216576

[119] CM O‟B Mueller A AGM S Arendt EK. Evaluation of the effect of fat replacer on quality of wheat bread. J Food Eng. (2003) 56:265–7. doi: 10.1016/S0260-8774(02)00266-2

[120] Akalin AS Karagozlu C Unal G. Rheological properties of reduced-fat and low-fat ice cream containing whey protein isolate and inulin. Eur Food Res Technol. (2008) 227:889–95. doi: 10.1007/s00217-007-0800-z

[121] Mansour EH Khalil AH. Characteristics of low-fat beef burger as influenced by various types of wheat fibers. Food Res Int. (1997) 30:199–205. doi: 10.1016/S0963-9969(97)00043-4

[122] Lipp M Anklam E. Review of cocoa butter and alternative fats for use in chocolate – part a. compositional data. Food Chem. (1998) 62:73–97. doi: 10.1016/S0308-8146(97)00160-X

[123] FAO. Food Safety and Quality: Synthetic and Processed Foods. Rome: Food and Agriculture Organization of the United Nations (2019).

[124] Warner JO. Artificial food additives: hazardous to long-term health? Arch Dis Child. (2024) 109:882–5. doi: 10.1136/archdischild-2023-326565, 38423749

[125] Shi S Wang Z Shen L Xiao H. Synthetic biology: a new frontier in food production. Trends Biotechnol. (2022) 40:781–803. doi: 10.1016/j.tibtech.2022.01.002, 35120749

[126] Tyagi A Kumar A Aparna SV Mallappa RH Grover S Batish VK. Synthetic biology: applications in the food sector. Crit Rev Food Sci Nutr. (2016) 56:1777–89. doi: 10.1080/10408398.2013.782534, 25365334

[127] Rodrigues RC Pereira HS Senra RL de Oliveira Barros Ribon A de Oliveira Mendes TA. Understanding the emerging potential of synthetic biology for food science: achievements, applications and safety considerations. Food Chem Adv. (2023) 3:100476. doi: 10.1016/j.focha.2023.100476

[128] Arun KB Anoopkumar AN Sindhu R Binod P Aneesh EM Madhavan A . Synthetic biology for sustainable food ingredients production: recent trends. Syst Microbiol Biomanuf. (2023) 3:137–49. doi: 10.1007/s43393-022-00150-3

[129] Kennedy K MadlerA NC Alcaine S. Advancing synthetic biology in the dairy industry: innovations, applications, and policy implications. J Dairy Sci. (2025) 109:83–91. doi: 10.3168/jds.2025-27456, 41176263

[130] UNEP. Food Systems and Natural Resources. Nairobi: United Nations Environment Programme (2021).

[131] Rugji JE Taşçı Z Musa F Hamadani L Gündemir A Siddiqui MG. Utilization of AI–reshaping the future of food safety, agriculture and food security–a critical review. Crit Rev Food Sci Nutr. (2025) 65:5136–80. doi: 10.1080/10408398.2024.2430749 39644464

[132] Wang Y Zhao J Jiang L Zhang L Raghavan V Wang J. A comprehensive review on novel synthetic foods: potential risk factors, detection strategies, and processing technologies. Compr Rev Food Sci Food Saf. (2024) 23:e13371. doi: 10.1111/1541-4337.13371, 38853463

[133] Hassani NEAE Baraket N Alem AC. Recent advances in natural food preservatives: a sustainable solution for food safety and shelf -life extension. J Food Meas Charact. (2025) 19:293–315. doi: 10.1007/s11694-024-02969-x

[134] Fadiji T Rashvand M Daramola MO Iwarere SA. A review on antimicrobial packaging for extending the shelf life of food. Processes. (2023) 11:590. doi: 10.3390/pr11020590

[135] Potter NN Hotchkiss JH. Food Science. 5th ed. New York: Springer (2012).

[136] Fellows PJ. Food Processing Technology: Principles and Practice.UK: Woodhead Publishing (2017).

[137] Fardet A. Food health potential is primarily due to its matrix structure: then nutrient composition. J Nutr. (2015) 145:613–20. doi: 10.15406/jnhfe.2014.01.00031

[138] Muncke J Touvier M Trasande L Scheringer M. Health impacts of exposure to synthetic chemicals in food. Nature Med. (2025) 31:1431–43. doi: 10.1038/s41591-025-03697-5, 40379996 PMC12442484

[139] Chassaing B Koren O Goodrich JK Poole AC Srinivasan S Ley RE . Dietary emulsifiers impact the mouse gut microbiota promoting colitis and metabolic syndrome. Nature. (2015) 519:92–6. doi: 10.1038/nature14232, 25731162 PMC4910713

[140] Çakmakçı S Polatoğlu B Çakmakçı R. Foods of the future: challenges, opportunities, trends, and expectations. Foods. (2024) 13:2663. doi: 10.3390/foods13172663, 39272427 PMC11393958

[141] Ramírez Rojas AA Swidah R Schindler D. Microbes of traditional fermentation processes as synthetic biology chassis to tackle future food challenges. Front Bioeng Biotechnol. (2022) 10. doi: 10.3389/fbioe.2022.982975 PMC9523148 36185425

[142] Chamodi KKD Vu NT Domingos JA Loh JY. Cellular solutions: evaluating single-cell proteins as sustainable feed alternatives in aquaculture. Biology. (2025) 14:764. doi: 10.3390/biology14070764, 40723325 PMC12292964

[143] Google Patents (2014). Additional Patent and Report Citations US Patent US20140356507A1. Plant-Based Egg Substitute and Method of Manufacture. Google Patents. Available online at: https://patents.google.com/patent/US20140356507A1/en (Accessed May 04, 2026).

